# Autophagy of germ-granule components, PGL-1 and PGL-3, contributes to DNA damage-induced germ cell apoptosis in *C*. *elegans*

**DOI:** 10.1371/journal.pgen.1008150

**Published:** 2019-05-24

**Authors:** Hyemin Min, Yong-Uk Lee, Yhong-Hee Shim, Ichiro Kawasaki

**Affiliations:** Department of Bioscience and Biotechnology, Konkuk University, Seoul, Republic of Korea; Harvard Medical School, UNITED STATES

## Abstract

Germ granules, termed P granules in nematode *C*. *elegans*, are the germline-specific cytoplasmic structures widely observed from worms to humans. P granules are known to have critical functions for postembryonic germline development likely through regulating RNA metabolism. They are localized at the perinuclear region of germ cells during most of the developmental stages. However, the biological significance of this specific localization remains elusive. PGL-1 and PGL-3, the defining components of P granules, were shown to be lost from the perinuclear region prior to germ cell apoptosis. Furthermore, this loss was shown to be significantly enhanced upon DNA damage. Here, we show that the removal of PGL-1 and PGL-3 from the perinuclear region following UV-induced DNA damage is significantly reduced in autophagy mutants. Autophagy was previously shown to be required for DNA damage-induced germ cell apoptosis. We show that the apoptosis defect of autophagy mutants is bypassed by depletion of *pgl-1* or *pgl-3*. These findings are consistent with time-lapse observations of LGG-1 foci formation, showing that autophagy is activated following UV irradiation and that maximal accumulation of LGG-1 foci occurs before PGL-1 removal. We also show that some of the autophagy genes are transcriptionally activated following UV irradiation by CEP-1, the worm p53-like protein. Taken together, our results indicate that autophagy is required to remove the major P granule components, PGL-1 and PGL-3, and that their removal is required for the full induction of DNA damage-induced germ cell apoptosis. Our study contributes to a better understanding of germ cell apoptosis, a process that leads to the elimination of the vast majority of germ cells in various animals from worms to mammals.

## Author summary

*C*. *elegans* provides a prime model for studying evolutionarily conserved biological mechanisms that control development and physiology. One of the conserved features of germ cells is the presence of germ granules, the germline-specific cytoplasmic structures observed in various organisms from worms to humans. P granules, the *C*. *elegans* germ granules, have critical functions for its postembryonic germline development. We previously reported that PGL-1 and PGL-3, the defining components of P granules, are lost from germ cells prior to germ cell apoptosis, and that this loss is significantly enhanced upon DNA damage. Here, we show that removal of PGL-1 and PGL-3 from germ cells following DNA damage is significantly reduced in autophagy mutants. Furthermore, the failure of autophagy mutants to increase germ cell apoptosis upon DNA damage is significantly recovered by depletion of *pgl-1* or *pgl-3*. We also show that autophagy, as measured by LGG-1 foci formation, is induced following DNA damage in adult hermaphrodite gonads in PGL-1, PGL-3, and CEP-1, the worm p53-like protein, dependent manner. Taken together, our results indicate that DNA damage activates autophagy through CEP-1 to remove PGL-1 and PGL-3 from germ cells, which contributes to fully induce germ cell apoptosis upon DNA damage in *C*. *elegans* adult hermaphrodites.

## Introduction

During mammalian germline development, the vast majority of germ cells are eliminated by apoptosis [1–5]. Studies in mouse models demonstrated that germ cell apoptosis following DNA damage requires the activities of the DNA damage response pathway and the p53 family members [6, 7]. The majority of germ cell apoptosis occurs during the pachytene stage in meiotic prophase I, in which failure of meiotic recombination and perturbation in chromosome structure are detected. The *Caenorhabditis elegans* germ line provides a model system to study the apoptotic elimination of germ cells [8]. In *C*. *elegans*, the germ cell apoptosis is restricted to female germ cells residing in the late pachytene stage. A basal level of germ cell apoptosis termed “physiological germ cell apoptosis” can be observed under standard culturing conditions [8]. Upon DNA damage or meiotic recombination failure, the germ cell apoptosis is further induced [9]. This type of germ cell apoptosis is called “DNA damage-induced germ cell apoptosis”. As does somatic programmed cell death, all types of germ cell apoptosis require the activities of both CED-4/Apaf-1-like adaptor protein and CED-3 caspase for the execution [10, 11]. Germ cell apoptosis is also negatively regulated by CED-9, an anti-apoptotic protein homologous to Bcl-2 [8, 9]. DNA damage-induced germ cell apoptosis in *C*. *elegans* requires the activation of multiple proteins involved in the DNA damage checkpoint pathway [9], including CEP-1, the sole *C*. *elegans* homolog of the mammalian p53 tumor suppressor family composed of p53, p63, and p73 [12]. DNA damage-induced germ cell apoptosis also requires the two pro-apoptotic BH3-only proteins, EGL-1 and CED-13, which are thought to promote apoptosis by directly antagonizing CED-9 [9, 13, 14]. EGL-1 and CED-13 are not required for physiological germ cell apoptosis.

Germ granules are germline-specific non-membrane-bound ribonucleoprotein organelles, which are observed in various animals from worms to humans [15, 16]. Germ granules are considered to play pivotal roles in the formation or maintenance of germ cells. In *C*. *elegans*, germ granules are also called P granules [17]. P granules are considered to regulate RNA metabolism of germ cells; most of the protein components so far identified contain RNA-binding motifs [18, 19]. In germ cells, P granules are localized at the cytoplasmic side of the nuclear envelope by associating with clusters of nuclear pores during most of the developmental stages [20–23]. It was postulated that P granules localize to the perinuclear region to control the transport of proteins and mRNAs between the nucleus and the cytoplasm [24, 25]. In previous studies, we identified PGL-1 and PGL-3 as the major constitutive components of P granules [26, 27]. The presence of PGL-1 and PGL-3, especially that of PGL-1, is essential for the functions of, and assembly of other components to, P granules [28–30]. In this study, we use PGL-1 and PGL-3 as markers for intact P granules. In *C*. *elegans* wild-type adult hermaphrodite gonads, although the majority of germ cells contain both PGL-1 and PGL-3 at their nuclear periphery as constitutive components of P granules, a few PGL-absent germ cells are also constantly observed in the pachytene region of the gonads under physiological conditions [21]. In a previous study, we found that the number of PGL-absent germ cells is significantly increased following UV irradiation [31]. In addition, under both physiological and DNA-damaged conditions, gonadal sheath cells selectively engulfed germ cells lacking PGL proteins, indicating that PGL-depleted germ cells are apoptotic cells and that the removal of PGL-1 and PGL-3 from germ cells occurs concomitant with germ cell apoptosis [31]. PGL-depleted germ cells are selectively committed to apoptosis because these cells do not efficiently retain SIR-2.1 in the nucleus [31]. SIR-2.1 is a *C*. *elegans* homolog of the Sirtuin family, and the translocation of SIR-2.1 from the nucleus to the cytoplasm has been correlated with the induction of germ cell apoptosis upon DNA damage [32].

**Fig 1:**
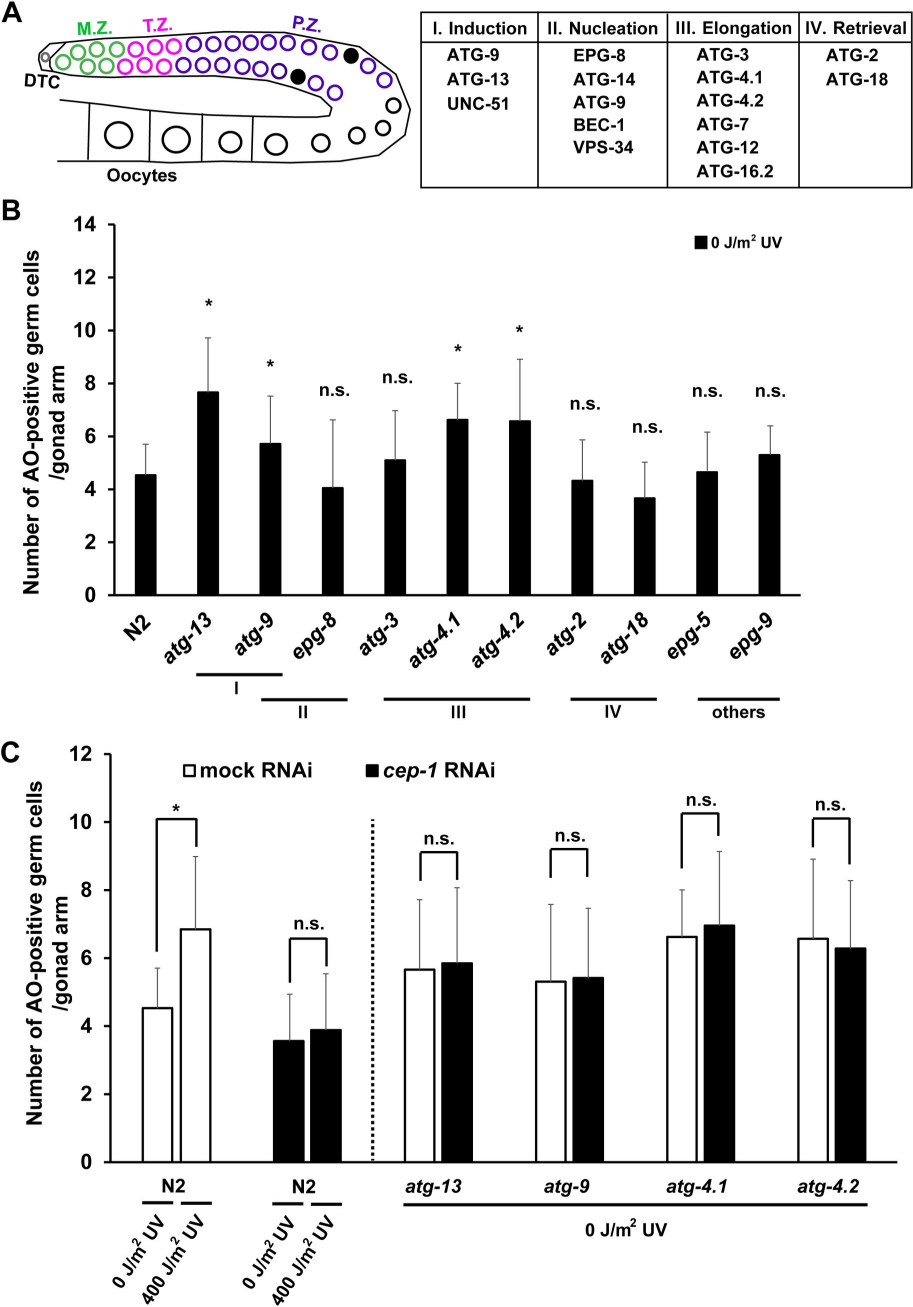
Some autophagy mutants showed higher physiological germ cell apoptosis than wild type, although neither DNA damage checkpoint pathway nor autophagy was activated. (A) Left panel: Schematic diagram of a gonad arm in *C*. *elegans* adult hermaphrodite. In the gonad arm, germ cells undergo mitotic proliferation at the distal “mitotic zone” (M.Z., green circles) under the control of a distal tip cell (DTC, a gray circle). During their passage through the “transition zone” (T.Z., pink circles), the germ cells stop dividing and initiate meiosis. As a result, germ cells at the pachytene stage in meiotic prophase I are accumulated in the “pachytene zone” (P.Z., purple circles). Apoptotic germ cells (black filled circles) are mostly observed in the late pachytene zone (P.Z.) near the loop of the gonad arm. The more proximal region of the gonad arm is filled with developing oocytes (black open circles). Right panel: The four distinct steps and relevant *C*. *elegans* autophagy gene products required for autophagosome formation. (B) Mean ± s.d. number of acridine orange (AO)-positive apoptotic germ cells per gonad arm in wild-type N2 and respective autophagy mutant hermaphrodites under physiological conditions (0 J/m^2^ UV). Acridine orange staining was performed at the adult (48 h post the L4) stage. Statistical significance was calculated using Student’s *t*-test and one-way ANOVA. n.s., *p* > 0.05 and *, *p* < 0.05 against N2. Number of analyzed gonads, n ≥ 35 for all the strains. (C) Mean ± s.d. number of acridine orange (AO)-positive apoptotic germ cells per gonad arm in N2, *atg-13(bp414)*, *atg-9(bp564)*, *atg-4*.*1(bp501)*, and *atg-4*.*2(tm3948)* mutant hermaphrodites with (black bars) or without (white bars) RNAi depletion of *cep-1* under physiological (0 J/m^2^ UV) and DNA-damaged (400 J/m^2^ UV) conditions. Acridine orange staining was performed at the adult (48 h post the L4) stage. Statistical significance was calculated using Student’s *t*-test and one-way ANOVA. n.s., *p* > 0.05 and *, *p* < 0.05. Number of analyzed gonads, n ≥ 35 for all the strains in respective conditions.

Macroautophagy (hereafter referred to as autophagy) is a ubiquitous intracellular degradation process conserved among eukaryotes including *C*. *elegans* [33, 34]. Autophagy sequesters cytoplasmic materials including organelles into a double-membrane vesicle termed autophagosome, which subsequently fuses with the lysosome to degrade the sequestered materials. The autophagic process can be dissected into several distinct steps, which include (1) induction, (2) cargo selection and packaging, (3) vesicle nucleation, (4) vesicle expansion and completion, (5) retrieval of autophagy proteins from vesicle, and (6) vesicle targeting, docking, and fusion with the lysosome [35]. Autophagy-related genes or *Atg* genes that act at respective autophagic steps have been identified mainly through genetic screens using yeast *Saccharomyces cerevisiae*. The *C*. *elegans* genome has orthologs for many of the yeast *Atg* genes [34]. It has been shown that four enzymatic complexes are involved in the formation of autophagosome (Fig 1A table) [34]. A serine/threonine protein kinase complex, which includes UNC-51 and ATG-13, induces autophagic activity. A class III phosphatidylinositol 3-kinase complex, which includes BEC-1 and VPS-34, acts for vesicle nucleation. Two ubiquitin-like conjugation pathways, including ATG-3, ATG-4, ATG-7, and LGG-1, bring about vesicle expansion and completion. Furthermore, a protein retrieval system, including ATG-2, ATG-9, and ATG-18, recycles autophagy proteins. In addition, some of the EPG (Ectopic P Granules) gene products, including EPG-2, EPG-11, and SEPA-1, are involved in cargo selection and packaging [36–38]. Autophagy is involved in multiple biological processes during *C*. *elegans* development [39, 40]. For example, autophagy selectively degrades sperm-derived paternal mitochondria and membranous organelles in newly fertilized embryos [41–43]. Autophagy is also required for the survival of newly hatched L1 larvae upon starvation [44], for the development of dauer larvae, and for lifespan extension [45]. Autophagy is further required to maintain the number of mitotically dividing germ cells in the distal region of gonads during larval development [46]. Furthermore, autophagy modulates several miRNA-mediated processes by downregulating components of RNA-induced silencing complex [47]. Notably, in relation to this study, it was reported that autophagy mediates the degradation of PGL-1 and PGL-3 that are missegregated to somatic blastomeres during embryogenesis [48].

Autophagy and apoptosis are often simultaneously activated during development and in response to stress [49–51]. However, molecular mechanisms that link the two degradation processes are still elusive. It was previously reported that loss of BEC-1, the *C*. *elegans* ortholog of Atg6/Vps30/Beclin 1, a key regulator of autophagy, increased the number of apoptotic cells in both the soma and the germ line [52]. BEC-1 was also shown to physically interact with the anti-apoptotic protein CED-9 [52]. Therefore, BEC-1 may also function as a regulator of apoptosis by interacting with CED-9. However, this apparent increase in the number of apoptotic germ cells in BEC-1-depleted hermaphrodite gonads could have been caused by a clearance defect of engulfed germ cell corpses in somatic gonadal sheath cells rather than by a *bona fide* increase in the number of germ cells undergoing apoptosis, because BEC-1 was also shown to be required for endocytosis in various somatic cells including the sheath cells [53]. Furthermore, depletion of another key endocytosis regulator VPS-34, the class III phosphatidylinositol 3-kinase that associates with BEC-1, also caused an apparent increase in the number of apoptotic germ cells under physiological conditions [53]. Therefore, further analysis is required to clarify whether BEC-1 indeed functions as a regulator that links autophagy to apoptosis. Another possible link between autophagy and apoptosis was reported to occur during DNA damage-induced germ cell apoptosis. That is, it was shown that autophagy is required for the full induction of germ cell apoptosis upon DNA damage [54]. However, it has not been elucidated how autophagy amplifies germ cell apoptosis following DNA damage.

Here, we show that some of the autophagy genes are transcriptionally upregulated following UV-induced DNA damage, and that this upregulation requires CEP-1. We show that DNA damage-induced autophagy removes PGL-1 and PGL-3 from a substantial number of germ cells, which leads to increase in the level of germ cell apoptosis. Our results suggest the presence of a novel mechanism that links autophagy to apoptosis, which is required for efficient induction of germ cell apoptosis following DNA damage.

## Results

### Some autophagy mutants show higher physiological germ cell apoptosis than wild type, although neither DNA damage checkpoint pathway nor autophagy is activated

To examine a possible link between autophagy and germ cell apoptosis, we first examined the levels of germ cell apoptosis in various autophagy mutants under physiological conditions (Fig 1). Apoptotic germ cells are observed in the pachytene region of adult hermaphrodite gonads (Fig 1A). To detect apoptotic germ cells, we used acridine orange (AO) vital staining as previously described [31, 55]. Because four distinct steps are involved in the formation of autophagosome (Fig 1A) [33, 34], we included the following homozygous viable autophagy mutants, which function in respective autophagic steps, in the apoptosis analysis: *atg-13(bp414)* and *atg-9(bp564)* functioning in the induction step (I), *epg-8(bp251)* in the nucleation step (II), *atg-3(bp412)*, *atg-4*.*1(bp501)*, and *atg-4*.*2(tm3948)* in the elongation step (III), *atg-2(bp576)* and *atg-18(gk378)* in the retrieval step (IV), and *epg-5(tm3425)* and *epg-9(bp320)*, which function in other steps (Fig 1A; S1B Fig). We found that four of the autophagy mutants, *atg-13(bp414)*, *atg-9(bp564)*, *atg-4*.*1(bp501)*, and *atg-4*.*2(tm3948)*, showed higher levels of germ cell apoptosis than wild-type N2 under physiological conditions (Fig 1B, *p* < 0.05). Physiological germ cell apoptosis can be increased by ectopic activation of DNA damage checkpoint pathway [56], and this signal is mediated by CEP-1, the sole *C*. *elegans* homolog of the mammalian p53 tumor suppressor family [12, 13]. Therefore, we tested whether the increased physiological germ cell apoptosis in the four autophagy mutants is suppressed by depletion of CEP-1. We found that although an increase in germ cell apoptosis in wild-type N2 following UV irradiation was significantly suppressed by RNAi depletion of *cep-1*, increased physiological germ cell apoptosis in the four autophagy mutants was not reduced to the N2 level after RNAi depletion of *cep-1* (Fig 1C). Therefore, we consider that the increase in physiological germ cell apoptosis in these autophagy mutants was not caused by ectopic activation of DNA damage checkpoint pathway. To evaluate whether there is a direct correlation between the levels of physiological germ cell apoptosis and the levels of autophagy activity in these autophagy mutants, we quantified the number of LGG-1 foci in the pachytene region of their gonads after immunostaining with anti-LGG-1 antibody (S1A Fig). LGG-1, the *C*. *elegans* ortholog of ATG8/LC3, is a commonly used autophagy marker, which forms cytoplasmic foci when autophagy is activated [34]. We found that none of the autophagy mutants we examined formed any LGG-1 foci in the pachytene region of their gonads under physiological conditions (S1C Fig). This result indicates that the level of physiological germ cell apoptosis in autophagy mutants is not directly regulated by their autophagy activity. It was previously reported that apparent increase in the level of physiological germ cell apoptosis after depletion of *bec-1* or *vps-34* was more likely caused by a clearance defect of engulfed germ cell corpses in somatic gonadal sheath cells rather than by a *bona fide* increase in germ cell apoptosis [53]. Therefore, although some autophagy mutants show higher levels of germ cell apoptosis than wild type under physiological conditions, this is likely caused by a mechanism that is related to but different from autophagy.

### Failure of autophagy mutants to increase germ cell apoptosis following DNA damage is significantly restored by depletion of *pgl-1* or *pgl-3*

**Fig 2:**
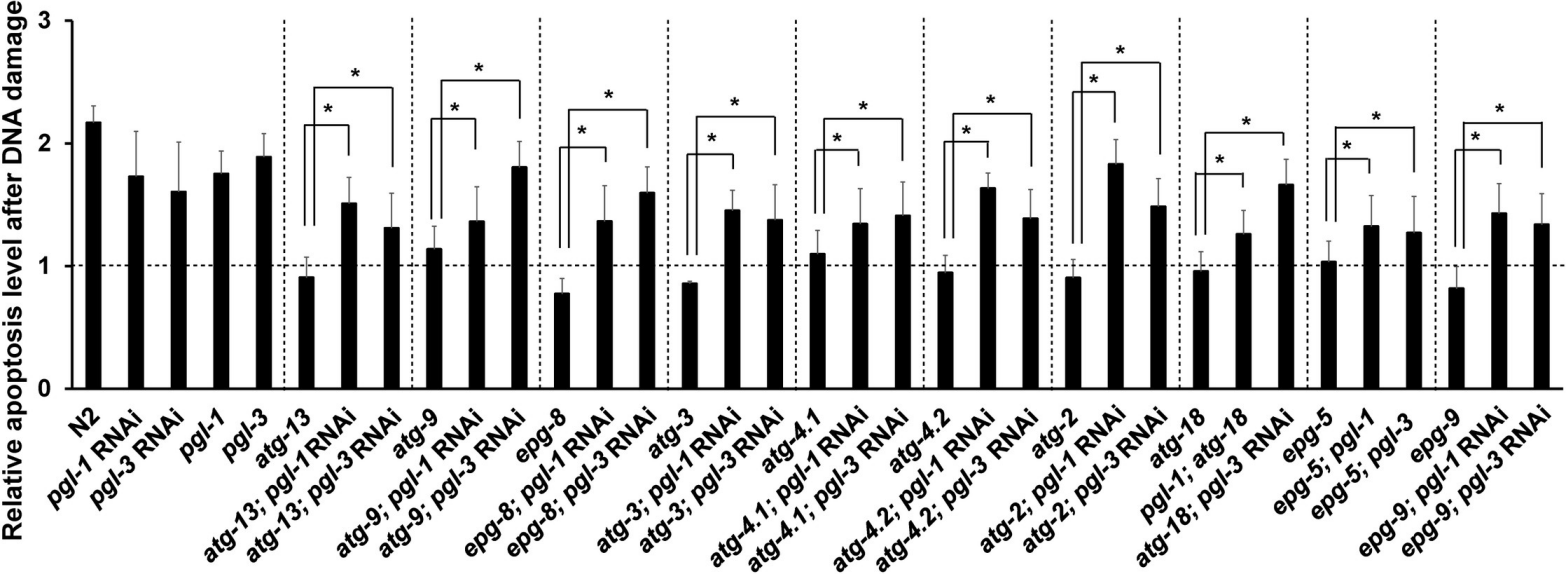
Failure of autophagy mutants to increase germ cell apoptosis following DNA damage was significantly rescued by depletion of *pgl-1* or *pgl-3*. Relative germ cell apoptosis levels of N2, *pgl-1(ct131)*, *pgl-3(bn104)*, and autophagy mutant hermaphrodites following DNA damage (400 J/m^2^ UV) compared with physiological (0 J/m^2^ UV) conditions. T-bars represent s.d. The same autophagy mutants as used in Fig 1B were examined. Respective autophagy mutants were combined or not combined with either *pgl-1(ct131)* or *pgl-3(bn104)* mutation (for *atg-18* and *epg-5* mutants), or treated or not treated with either *pgl-1* RNAi or *pgl-3* RNAi depletion (for N2 and the other autophagy mutants) before quantification of apoptotic germ cells, which was performed as in Fig 1. Statistical significance was calculated using Student’s *t*-test and one-way ANOVA. *, *p* < 0.05 compared with respective single autophagy mutants. Number of analyzed gonads, n ≥ 30 for all the strains in respective conditions.

It was previously reported that autophagy mutants failed to increase the level of germ cell apoptosis following DNA damage [54]. We confirmed this result (Fig 2). First, germ cell apoptosis was increased substantially following UV irradiation compared with non-irradiated conditions in wild-type N2, *pgl-1* mutant, and *pgl-3* mutant adult hermaphrodites, as previously described (Fig 2) [9, 10, 31]. Second, in contrast to N2, *pgl-1* mutant, and *pgl-3* mutant, the levels of germ cell apoptosis were not significantly changed following UV irradiation compared with non-irradiated conditions in respective single autophagy mutants, as previously reported (Fig 2) [54]. We further examined whether the levels of germ cell apoptosis in respective autophagy mutants are affected by depletion of *pgl-1* or *pgl-3* because of the following reasons. First, we previously found that removal of PGL-1 and PGL-3 from germ cells was significantly increased in N2 adult hermaphrodite gonads following UV irradiation (S2 Fig), and that this removal of PGL proteins contributed to increase germ cell apoptosis upon DNA damage [31]. Second, it was previously reported that autophagy mediates the degradation of PGL-1 and PGL-3 when they are missegregated to somatic blastomeres during embryogenesis [48]. We found that the levels of germ cell apoptosis were significantly increased following UV irradiation compared with non-irradiated conditions in all the autophagy mutants when *pgl-1* or *pgl-3* was simultaneously depleted by either RNAi or mutation (Fig 2, *p* < 0.05 compared with respective single autophagy mutants). Our results suggest that the failure of autophagy mutants to increase germ cell apoptosis following DNA damage is caused, at least in part, by their failure to remove PGL-1 and/or PGL-3 from germ cells upon DNA damage.

### Removal of PGL-1 and PGL-3 from germ cells following UV irradiation significantly decreases in autophagy mutant hermaphrodite gonads

**Fig 3:**
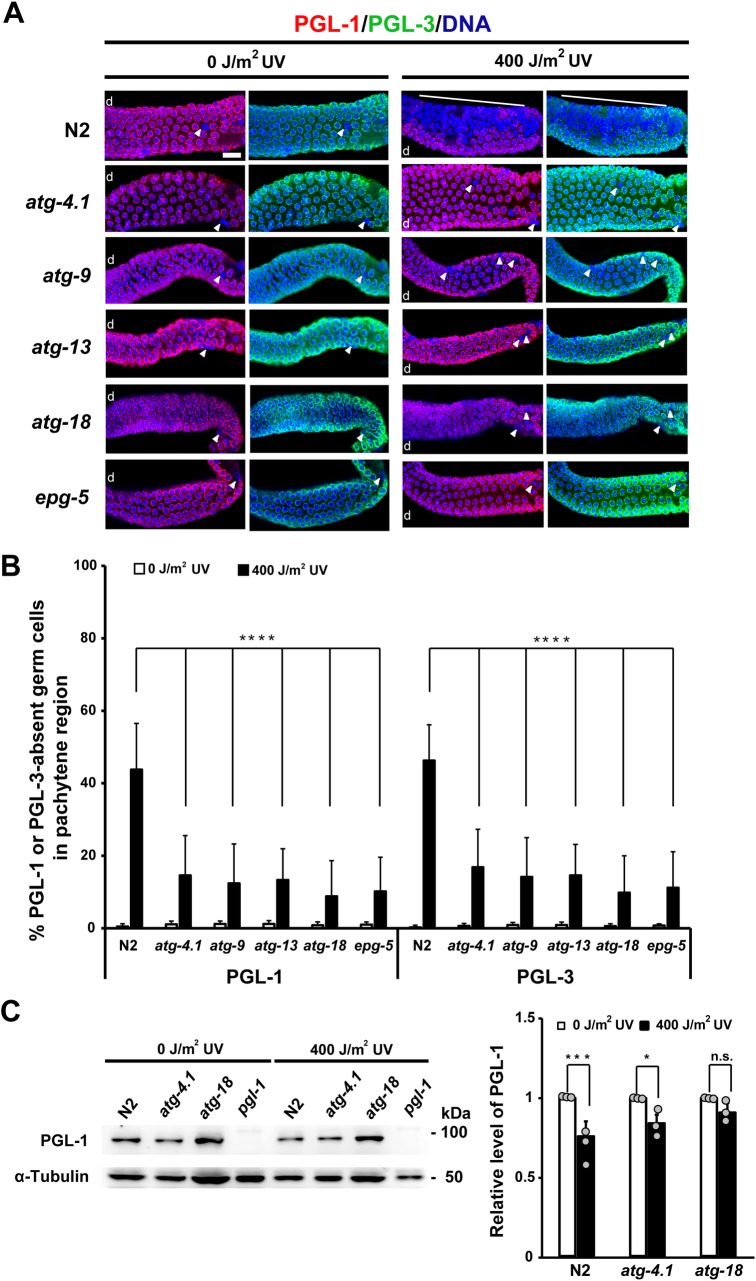
Removal of PGL-1 and PGL-3 from UV-irradiated or non-irradiated autophagy mutant hermaphrodite gonads. (A) Removal of perinuclearly localized PGL-1 (red) and PGL-3 (green) from germ nuclei (blue) in the pachytene region of adult hermaphrodite gonads from wild-type N2 and respective autophagy mutants, which were irradiated (400 J/m^2^) or not irradiated (0 J/m^2^) with UV. As autophagy mutants, *atg-4*.*1(bp501)*, *atg-9(bp564)*, *atg-13(bp414)*, *atg-18(gk378)*, and *epg-5(tm3425)* were examined. Arrowheads indicate a few germ nuclei that lack PGL proteins in respective gonad arms. White lines indicate clusters of PGL-depleted germ nuclei in the UV-irradiated N2 gonad arm. d, distal side of each gonad arm. Scale bar, 20 μm. (B) Mean ± s.d. percent of PGL-1 or PGL-3-absent germ cells (nuclei) among total germ cells (nuclei) in the pachytene region of adult hermaphrodite gonads in N2 and respective autophagy mutants with (400 J/m^2^, black bars) or without (0 J/m^2^, white bars) UV irradiation. Statistical significance was calculated using Student’s *t*-test. ****, *p* < 0.0001 against UV-irradiated N2. Number of analyzed gonads, n ≥ 30 for all the strains in respective conditions. (C) Western blot analysis of PGL-1 protein levels in N2, *atg-4*.*1(bp501)*, *atg-18(gk378)*, and *pgl-1(ct131)* adult hermaphrodites with (400 J/m^2^) or without (0 J/m^2^) UV irradiation. Whole worm protein extract obtained from ca. 100 adult hermaphrodites of each genotype was loaded per lane. *pgl-1* mutant lane was included to show the specificity of anti-PGL-1 antibody. Respective PGL-1 band intensities were normalized against those of α-Tubulin on the same lane. Then, the normalized “UV-irradiated” PGL-1 band intensity of each genotype was converted to a relative value (gray dots on black bars) compared to the normalized “non-irradiated” PGL-1 band intensity of the same genotype (gray dots on white bars, which were converted as value 1), as shown in the right graph with their mean ± s.d. values. These PGL-1 band intensity values were obtained from three independent western blot analyses. Statistical significance was calculated using Student’s *t*-test. n.s., *p* > 0.05. *, *p* < 0.05. ***, *p* < 0.001.

To examine whether the removal of PGL-1 and PGL-3 from germ cells upon DNA damage is indeed compromised in autophagy mutants, we irradiated or not irradiated adult hermaphrodites of N2 and several autophagy mutants with UV, and immunostained their dissected gonads with anti-PGL-1 and anti-PGL-3 antibodies (Fig 3). First, we confirmed that both PGL-1 and PGL-3 were removed from a large number of germ cells in the pachytene region of UV-irradiated N2 adult hermaphrodite gonads, in contrast to non-irradiated N2 hermaphrodite gonads (Fig 3A, white lines; also see S2 Fig). Second, we found that compared with N2, removal of PGL-1 and PGL-3 from germ cells following UV irradiation was significantly reduced in adult hermaphrodite gonads of all the autophagy mutants examined, including *atg-4*.*1(bp501)*, *atg-9(bp564)*, *atg-13(bp414)*, *atg-18(gk378)*, and *epg-5(tm3425)*, in which only a small number of germ cells lost PGL-1 and PGL-3 (Fig 3A, white arrowheads). The above observations were confirmed by the quantification of the number of PGL-absent germ cells in the pachytene region (Fig 3B). We further examined whether the protein level of PGL-1 in adult hermaphrodites is also reduced following UV irradiation in an autophagy activity-dependent manner, by western blot analysis of N2 and autophagy mutant adult hermaphrodites using anti-PGL-1 antibody (Fig 3C). We found that the protein level of PGL-1 was indeed significantly reduced in N2 adult hermaphrodites following UV irradiation compared to non-irradiated condition (Fig 3C, *p* < 0.001). In contrast, PGL-1 protein level was less significantly reduced in *atg-4*.*1(bp501)* adult hermaphrodites (*p* < 0.05) and not significantly reduced in *atg-18(gk378)* adult hermaphrodites (*p* > 0.05) following UV irradiation compared to non-irradiated condition (Fig 3C). These results indicate that at least a portion of PGL-1 protein is degraded following UV irradiation in an autophagy activity-dependent manner in adult hermaphrodites. Therefore, autophagy is most likely involved in the removal and/or degradation of PGL proteins in adult hermaphrodite gonads in response to DNA damage.

### Following UV irradiation, LGG-1 foci are formed in the pachytene region of gonads in hermaphrodites, but not in males

**Fig 4:**
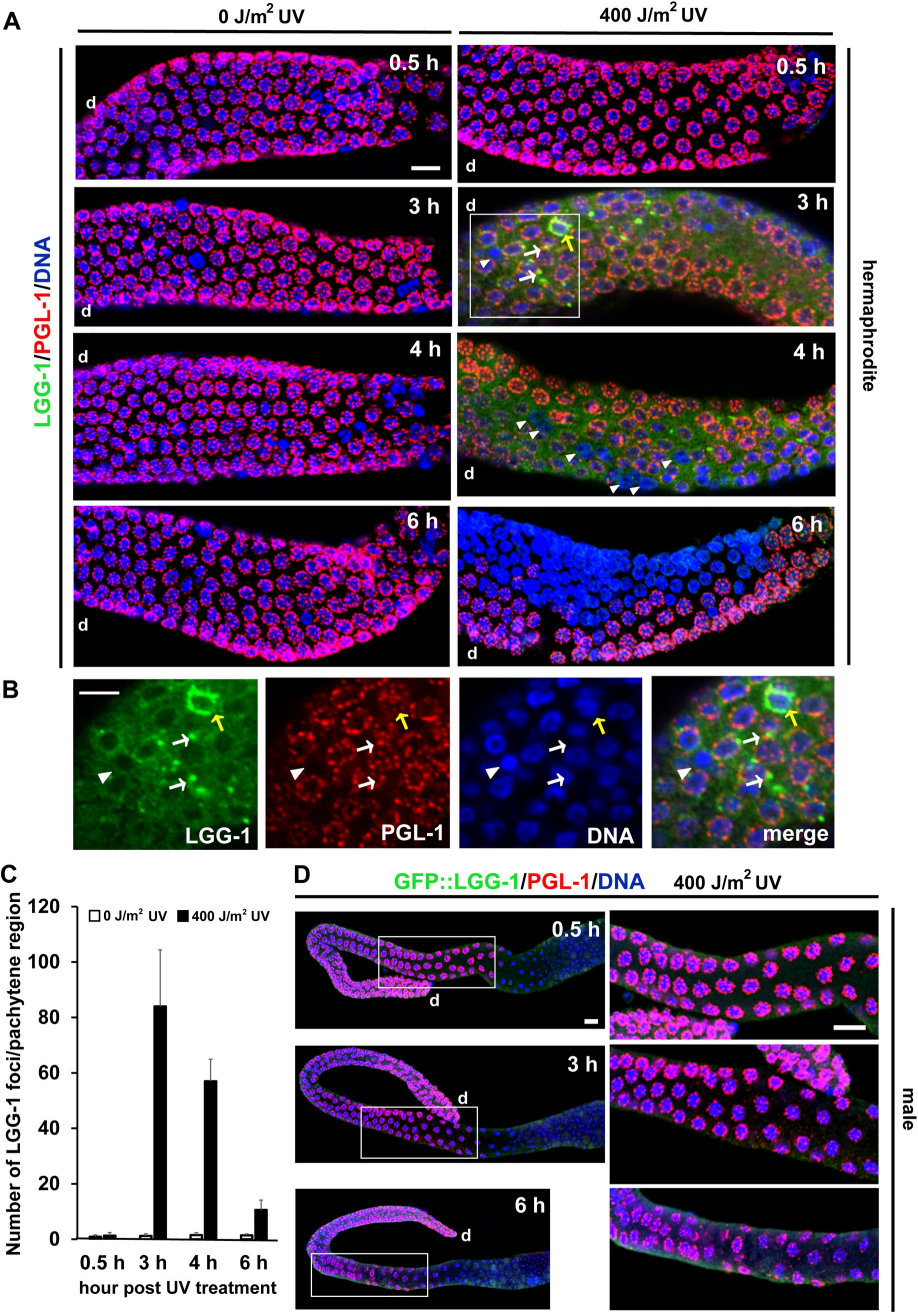
LGG-1 foci formation following UV irradiation in hermaphrodite and male gonads. (A) Confocal immunofluorescence images showing the time course of LGG-1 foci formation (green) and associated removal of PGL-1 (red) from germ nuclei (blue) in the pachytene region of N2 adult hermaphrodite gonads following 0 or 400 J/m^2^ of UV irradiation. More than 20 gonads were observed at respective time points under respective conditions. (0.5 h) Neither LGG-1 foci formation nor removal of PGL-1 was observed in the majority of germ cells in both the UV-irradiated and non-irradiated gonads. (3 h) A significant number of LGG-1 foci were observed in the cytoplasm and at the perinuclear region of germ cells (arrows in the inset), and PGL-1 started to disappear from some germ cells (an arrowhead in the inset) in the UV-irradiated gonad. The non-irradiated gonad appeared not different from the one at the 0.5 h time point. (4 h) The number of LGG-1 foci decreased compared to the 3 h time point, and PGL-1 was removed from a significant number of germ cells (arrowheads) in the UV-irradiated gonad. The non-irradiated gonad appeared not different from the ones at the 0.5 h and 3 h time points. (6 h) LGG-1 foci largely disappeared, whereas PGL-1 was lost from around 50% of germ cells (a cluster of blue germ nuclei) in the pachytene region of the UV-irradiated gonad. The non-irradiated gonad still appeared not different from the ones at the 0.5 h, 3 h, and 4 h time points. (B) The signals of LGG-1 (green), PGL-1 (red), and DNA (blue) in the inset (the area enclosed with a white square in the UV-irradiated 3 h gonad image) in (A) were enlarged and displayed separately along with their merged image for better appreciation of their localization and association in the area. The white arrows indicate LGG-1 foci that are located adjacent to PGL-1 granules. The yellow arrow indicates a cluster of LGG-1 foci that surround several PGL-1 granules. (C) Mean ± s.d. number of LGG-1 foci formed in the pachytene region of N2 adult hermaphrodite gonads at respective time points following 0 J/m^2^ (white bars) or 400 J/m^2^ (black bars) of UV irradiation. Number of analyzed gonads, n ≥ 20 for each time point and condition. (D) Confocal immunofluorescence images showing the equivalent time course of GFP::LGG-1 expression (green) and PGL-1 localization (red) in adult male gonads expressing a *pie-1* promoter-driven GFP::LGG-1 transgene following 400 J/m^2^ of UV irradiation. Number of observed gonads, n = 15 for each time point. Left column, the entire gonads. Right column, enlarged pachytene region of respective gonads. Neither LGG-1 foci formation nor PGL-1 removal was observed in the pachytene region of male gonads following UV irradiation through the observation (0–6 h). d, distal side of each gonad. Scale bars, 20 μm.

To directly examine whether autophagy is indeed activated following DNA damage in the pachytene region of adult hermaphrodite gonads, in which germ cell apoptosis takes place, we observed the formation of LGG-1 foci in the pachytene region in a time-course following UV irradiation (Fig 4). LGG-1 forms cytoplasmic foci when autophagy is activated [34]. We treated N2 or GFP::LGG-1 transgenic animals, which express GFP::LGG-1 in the germ line [41], with UV irradiation. Subsequently, a subset of these animals was collected every few hours, and their gonads were immunostained with either anti-LGG-1 [41] or anti-GFP antibody along with anti-PGL-1 antibody co-immunostaining and DNA counterstaining (Fig 4A, 4B and 4D). In both N2 and the GFP::LGG-1 transgenic adult hermaphrodites, we found that LGG-1 foci started to appear soon after UV irradiation, and the foci formation was maximal at 3 h after irradiation in the cytoplasm, and occasionally around the perinuclear region, of germ cells in the pachytene region of their gonads (Fig 4A and 4B, white and yellow arrows). The above observation was confirmed by the quantification of the number of LGG-1 foci formed in the pachytene region of gonads with or without UV irradiation (Fig 4C). The formation of LGG-1 foci in the cytoplasm of pachytene-stage germ cells following UV irradiation was also successfully observed by time-lapse live imaging of a hermaphrodite carrying an integrated *Ppie-1*::*GFP*::*lgg-1* transgene, after simultaneous depletion of *asp-10*, *vha-5*, and *vha-13* to suppress quick turnover of LGG-1 foci via reducing the activities of lysosomal enzymes (S3 Fig) [57]. We also observed that removal of PGL-1 from germ cells became prominent following the peak of LGG-1 foci formation, around 3–6 h after UV irradiation (Fig 4A and 4B, arrowheads). In contrast, in adult male gonads, neither UV-induced LGG-1 foci nor PGL-1 depletion was observed in the pachytene region, consistent with the absence of apoptosis in the male germ line (Fig 4D). Our results indicate that DNA damage induces autophagy in hermaphrodite pachytene-stage germ cells before the massive degradation of P granules becomes evident.

### PGL-1 and PGL-3 are required for the formation of LGG-1 foci following DNA damage in adult hermaphrodite gonads

**Fig 5:**
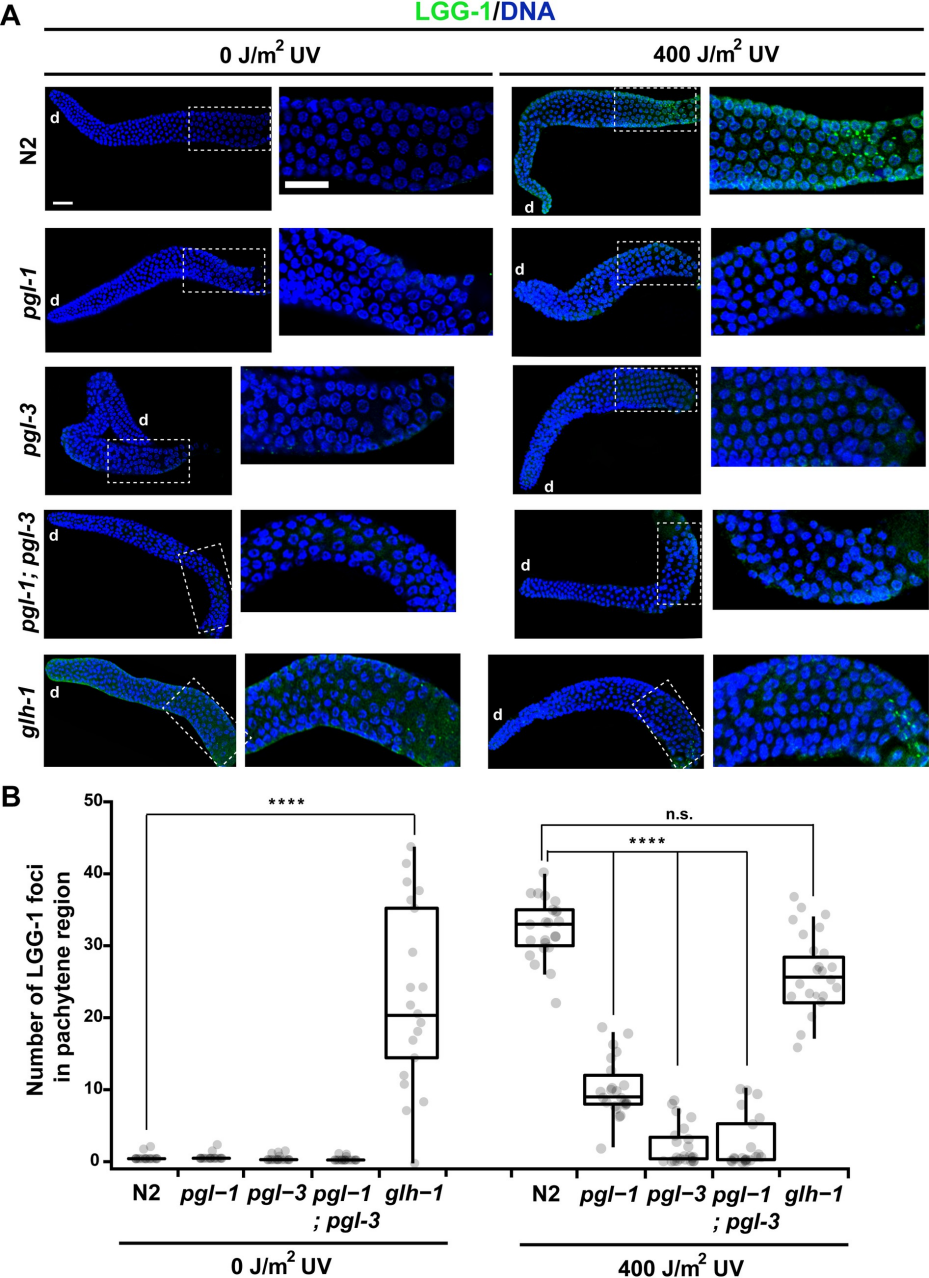
LGG-1 foci formation in P granule mutant hermaphrodite gonads with or without UV irradiation. (A) Wild-type N2, *pgl-1(ct131)* single, *pgl-3(bn104)* single, *pgl-1(ct131); pgl-3(bn104)* double, and *glh-1(ok439)* single mutant hermaphrodites were irradiated or not irradiated with 400 J/m^2^ of UV at 24 h after the L4 stage, collected at 3 h after the UV irradiation, and dissected and immunostained with anti-LGG-1 antibody (green) along with DNA counterstaining (blue). d, distal side of each gonad arm. Scale bars, 20 μm. Insets and their enlarged images show a proximal area of pachytene region in respective gonad arms. (B) Box-and-whisker plots depicting the number of LGG-1 foci formed in the pachytene region of gonad arms from N2 and respective mutants with or without 400 J/m^2^ of UV irradiation. Horizontal lines in respective boxes represent the median. Upper lines and lower lines extended from respective boxes represent 75% quartile and 25% quartile, respectively. Gray dots indicate numbers of LGG-1 foci formed in the pachytene region of respective gonad arms. Number of analyzed gonads, n ≥ 20 for all the strains in respective conditions. Statistical significance was calculated using Student’s *t*-test. ****, *p* < 0.0001 and n.s., *p* > 0.05 against N2.

We examined the levels of LGG-1 foci in various autophagy mutants following UV irradiation. We found that, in contrast to N2, all of the examined autophagy mutants failed to form LGG-1 foci efficiently in the pachytene region of their gonads following UV irradiation (S1A and S1C Fig). These results indicate that all these autophagy gene activities are required to form LGG-1 foci in the pachytene region of adult hermaphrodite gonads following DNA damage. We next asked whether the formation of LGG-1 foci following DNA damage requires PGL-1 and/or PGL-3. We therefore treated N2, *pgl-1* single, *pgl-3* single, and *pgl-1; pgl-3* double mutant hermaphrodites with UV irradiation, collected these animals at 3 h after UV irradiation, and immunostained their gonads with anti-LGG-1 antibody along with DNA counterstaining (Fig 5A). We found that the number of LGG-1 foci generated following UV irradiation significantly decreased in gonads of *pgl-1* single, *pgl-3* single, and *pgl-1; pgl-3* double mutant hermaphrodites compared with N2 hermaphrodite gonads (Fig 5A and 5B). Especially, in *pgl-3* single and *pgl-1; pgl-3* double mutant hermaphrodite gonads, only a very small number of LGG-1 foci were observed in the pachytene region following UV irradiation (Fig 5A and 5B). These results indicate that PGL-1 and PGL-3, especially PGL-3, are required to induce autophagy following DNA damage.

We also examined the formation of LGG-1 foci in *glh-1* mutant hermaphrodite gonads. GLH-1 is a member of another family of constitutive P granule components [58]. It was previously shown that in *glh-1* mutant germ cells, PGL-1 and PGL-3 are dissociated from the perinuclear region and dispersed to the cytoplasm [26, 27, 59]. It was also shown that germ cell apoptosis was more increased in *glh-1* mutant hermaphrodites than in N2 hermaphrodites under both physiological and DNA-damaged conditions as in *pgl-1* and *pgl-3* mutants [31]. We found that, in contrast to N2 hermaphrodite gonads, a substantial number of LGG-1 foci were persistently present in the pachytene region of *glh-1* mutant hermaphrodite gonads with or without UV irradiation (Fig 5A and 5B). These results suggest that subcellular localization of PGL-1 and PGL-3, that is, whether they localize at the nuclear periphery or they are dispersed to the cytoplasm, influences the formation of LGG-1 foci (see Discussion).

### SEPA-1 is not required for the autophagic removal of PGL-1 in adult hermaphrodite gonads

It was previously shown that SEPA-1 (Suppressor of Ectopic P granules in Autophagy mutants) functions as a bridging molecule that directly binds to both PGL-3 and the autophagy protein LGG-1 to mediate degradation of PGL-1 and PGL-3, which are missegregated to somatic blastomeres, during embryogenesis [48]. It was also reported that autophagic removal of PGL proteins from somatic blastomeres is impaired when *epg-11*, encoding an arginine methyltransferase that methylates PGL-1 and PGL-3, or *epg-2*, encoding a scaffold protein that associates with SEPA-1 aggregates, is mutated [60].

**Fig 6:**
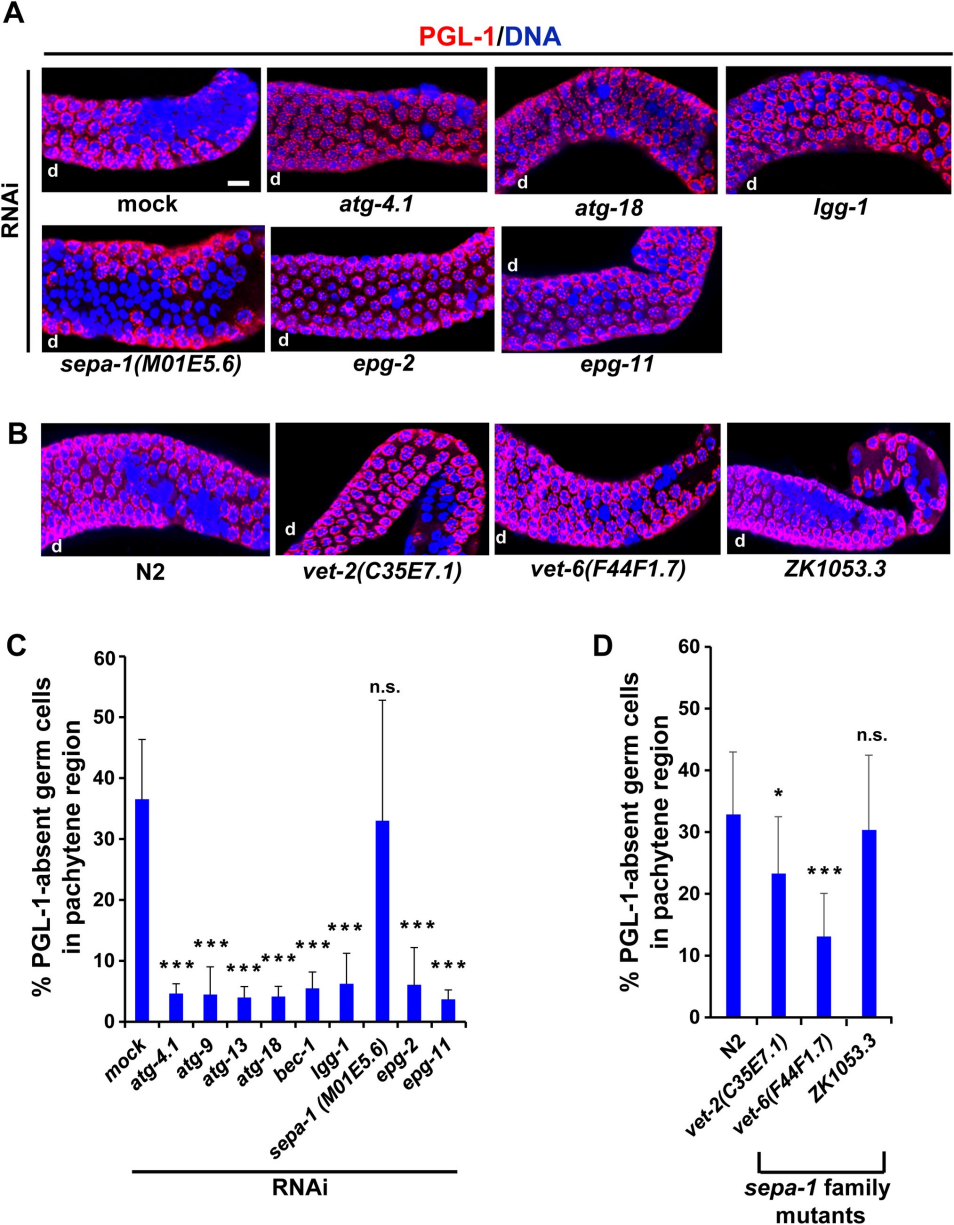
Removal of PGL-1 following UV irradiation in adult hermaphrodite gonads after depletion of autophagy-related genes. (A) Removal of PGL-1 (red) from germ nuclei (blue) following 400 J/m^2^ of UV irradiation in the pachytene region of N2 adult hermaphrodite gonads, in which respective autophagy-related genes, including *atg-4*.*1*, *atg-18*, *lgg-1*, *sepa-1*, *epg-2*, and *epg-11*, were depleted by RNAi. (B) Removal of PGL-1 (red) from germ nuclei (blue) following 400 J/m^2^ of UV irradiation in the pachytene region of N2 and *sepa-1* family mutant adult hermaphrodite gonads. As *sepa-1* family mutants, we examined *vet-2(ok1392)*, *vet-6(tm1226)*, and *zk1053*.*3(tm10601)* mutants, which have a deletion mutation in C35E7.1, F44F1.7, and ZK1053.3, respectively. d, distal side of each gonad arm. Scale bar, 20 μm. (C) Mean ± s.d. percent of PGL-1-absent germ cells (nuclei) in the pachytene region of N2 adult hermaphrodite gonads following 400 J/m^2^ of UV irradiation. These N2 hermaphrodites had been treated with RNAi to deplete respective autophagy-related genes. Statistical significance was calculated using Student’s *t*-test. ***, *p* < 0.001 and n.s., *p* > 0.05 against mock RNAi-treated N2 gonads. (D) Mean ± s.d. percent of PGL-1-absent germ cells (nuclei) in the pachytene region of N2 and *sepa-1* family mutant adult hermaphrodite gonads following 400 J/m^2^ of UV irradiation. Number of analyzed gonads, n ≥ 12 for all the strains in respective conditions. Statistical significance was calculated using Student’s *t*-test. *, *p* < 0.05, ***, *p* < 0.001, and n.s., *p* > 0.05 against N2 gonads.

To examine whether these activities are also required for autophagic removal of PGL proteins from germ cells in adult hermaphrodite gonads, we measured the extent of PGL-1 removal in UV-irradiated hermaphrodite gonads after RNAi depletion of the genes described above (Fig 6A and 6C). Because SEPA-1 is a member of a protein family consisting of 11 members [48], we also included homozygous viable deletion mutants of three genes, *vet-2* (C35E7.1), *vet-6* (F44F1.7), and ZK1053.3, which encode other members of SEPA-1 family, in this analysis (Fig 6B and 6D). As expected, we found that the RNAi depletion of *atg-4*.*1*, *atg-9*, *atg-13*, *atg-18*, *bec-1*, and *lgg-1*, which all served as positive controls, led to dramatically reduced PGL-1 removal compared with a mock RNAi control (Fig 6A and 6C, *p* < 0.001). The same result was observed upon *epg-2* and *epg-11* RNAi depletion (Fig 6A and 6C, *p* < 0.001), indicating that the scaffold protein EPG-2 and the arginine methyltransferase EPG-11 play critical roles during the autophagic removal of PGL-1 in adult hermaphrodite gonads as in somatic blastomeres. In contrast, RNAi depletion of *sepa-1* did not cause a significant reduction in PGL-1 removal (Fig 6A and 6C, *p* > 0.05). We confirmed that our treatment for RNAi depletion of *sepa-1* was effective because the same *sepa-1* RNAi treatment effectively phenocopied the *sepa-1* mutant phenotype [48]. That is, ectopic formation of PGL granules in somatic blastomeres of autophagy mutant embryos was efficiently suppressed by our *sepa-1* RNAi treatment (S4 Fig). We also examined the expression pattern of SEPA-1 using an integrated transgenic strain HZ455, in which *sepa-1*::*GFP* is expressed under the control of its own promoter (S5 Fig). We observed that SEPA-1::GFP was not expressed at a detectable level in gonadal germ cells in HZ455 adult hermaphrodites (S5 Fig). These results support the view that SEPA-1 does not play a critical role in the autophagic removal of PGL-1 in adult hermaphrodite gonads as opposed to its critical role in somatic blastomeres. On the other hand, among the three deletion mutants for other SEPA-1 family members, we found that *vet-2* and *vet-6* mutant hermaphrodites reduced the level of PGL-1 removal following UV irradiation compared with N2 hermaphrodites (Fig 6B and 6D, *p* < 0.05 for *vet-2* and *p* < 0.001 for *vet-6*). Furthermore, we found that the formation of LGG-1 foci following UV irradiation in the pachytene region was significantly reduced in *vet-6* mutant hermaphrodite gonads compared with N2 gonads (S6A and S6B Fig, *p* < 0.001). Therefore, in place of SEPA-1, other members of SEPA-1 family, such as VET-2 and/or VET-6, may function as a bridging molecule, or another class of protein may function as a bridging molecule, to link PGL proteins with the core autophagic machinery. In summary, our results suggest that the autophagic machinery that removes PGL proteins from gonadal germ cells largely overlaps with the machinery removing PGL proteins from somatic blastomeres except for the requirement of SEPA-1.

### CEP-1, but not EGL-1, is required to activate autophagy following DNA damage for the removal of PGL proteins in adult hermaphrodite gonads

**Fig 7:**
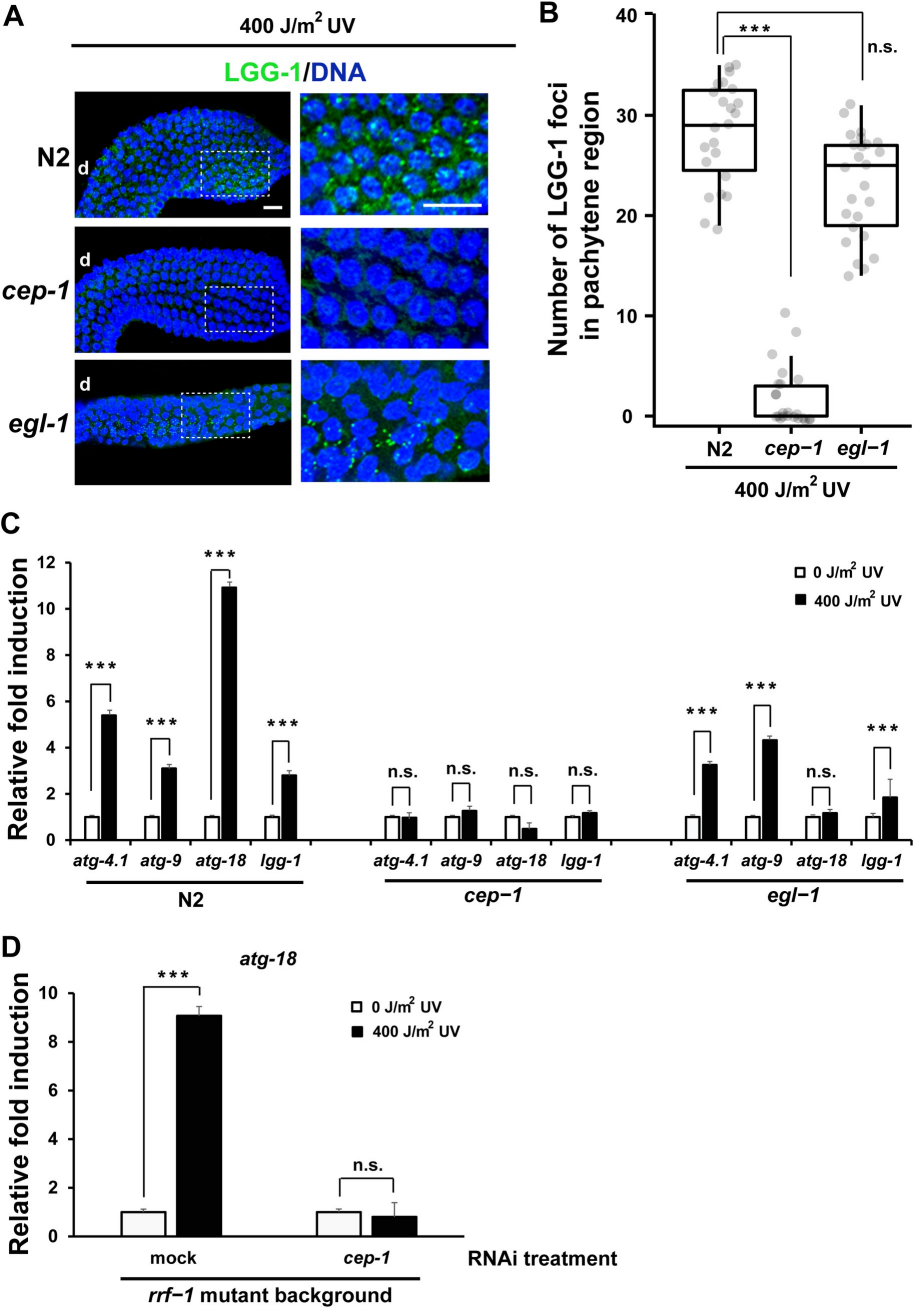
Several autophagy genes were transcriptionally up-regulated by CEP-1 following UV irradiation. (A) Formation of LGG-1 foci (green) at 3 h after 400 J/m^2^ of UV irradiation in the pachytene region of N2, *cep-1(gk138)*, and *egl-1(n487)* adult hermaphrodite gonads. Blue, DNA counterstaining. d, distal side of each gonad arm. Scale bars, 20 μm. Insets and their enlarged images show a proximal area of pachytene region in respective gonad arms. (B) Box-and-whisker plots depicting the number of LGG-1 foci formed in the pachytene region of gonad arms from N2, *cep-1(gk138)*, and *egl-1(n487)* adult hermaphrodites following 400 J/m^2^ of UV irradiation. The box-and-whisker plots are drawn as in Fig 5B. Number of analyzed gonads, n ≥ 15 for all the strains. Statistical significance was calculated using Student’s *t*-test. ***, *p* < 0.001 and n.s., *p* > 0.05 against N2 gonad arms. (C) Fold induction of mRNA levels for autophagy genes, *atg-4*.*1*, *atg-9*, *atg-18*, and *lgg-1*, following 400 J/m^2^ of UV irradiation (black) compared with non-irradiated (0 J/m^2^ UV) conditions (white) in N2, *cep-1(gk138)*, and *egl-1(n487)* adult hermaphrodites. (D) Fold induction of mRNA level for *atg-18* gene following 400 J/m^2^ of UV irradiation (black) compared with non-irradiated (0 J/m^2^ UV) conditions (white) in *rrf-1(pk1417)* adult hermaphrodites, which were treated or not treated with *cep-1* RNAi depletion. The mRNA levels of autophagy genes in respective strains under respective conditions were determined through 3 to 4 independent qRT-PCR experiments using the mRNA level of *act-1* in each sample as an internal control for normalization. Relative fold induction in mRNA level following UV irradiation (black) compared with non-irradiated conditions (white) was presented by converting the latter (white) values as 1. T-bars represent s.d. About 1200 adult hermaphrodite individuals (triplicates of 400 individuals) were used to prepare total RNA from respective strains in respective conditions in these qRT-PCR experiments. Statistical significance was calculated using Student’s *t*-test. ***, *p* < 0.001 and n.s., *p* > 0.05.

In a previous study, we found that the removal of PGL-1 from germ cells following DNA damage was significantly reduced in *cep-1*, but not *egl-1*, mutant gonads [31]. Furthermore, the failure to increase germ cell apoptosis following DNA damage was significantly rescued by a *pgl-1* mutation in *cep-1*, but not *egl-1*, mutant hermaphrodites [31]. Given that the apoptosis defect of both *cep-1* and autophagy mutants was rescued by depletion of PGL-1, we tested the possibility that CEP-1 and autophagy act in the same pathway. We first tested whether autophagy induction requires the activity of CEP-1 and/or EGL-1. We found that the formation of LGG-1 foci following UV irradiation was significantly reduced in the pachytene region of *cep-1*, but not *egl-1*, mutant hermaphrodite gonads compared with N2 hermaphrodite gonads (Fig 7A and 7B; also see S7 Fig for their LGG-1 foci formation without UV irradiation). These results indicate that the activity of CEP-1, but not EGL-1, is required to activate autophagy in the pachytene region following DNA damage to facilitate the removal of PGL proteins in adult hermaphrodite gonads.

To examine whether CEP-1 transcriptionally activates autophagy genes following DNA damage, we measured mRNA levels of several autophagy genes including *atg-4*.*1*, *atg-9*, *atg-18*, and *lgg-1* in N2, *cep-1* mutant, and *egl-1* mutant hermaphrodites under both non-irradiated and UV-irradiated conditions using qRT-PCR (Fig 7C). We found that the mRNA levels of these four genes significantly increased following UV irradiation in N2, but not *cep-1* mutant, hermaphrodites, indicating that CEP-1 is required for transcriptional activation of all the four autophagy genes following UV irradiation (Fig 7C). In *egl-1* mutant hermaphrodites, the mRNA levels of *atg-4*.*1*, *atg-9*, and *lgg-1* genes increased but the mRNA level of *atg-18* gene failed to increase following UV irradiation (Fig 7C). These results indicate that EGL-1 is not required for transcriptional activation of several autophagy genes including *atg-4*.*1*, *atg-9*, and *lgg-1* following UV irradiation. On the other hand, *atg-18* gene seems to require the activities of both CEP-1 and EGL-1 for the transcriptional activation following UV irradiation. To examine whether CEP-1 functions in the germ line for the transcriptional activation of autophagy genes, we measured the mRNA level of *atg-18* gene in *rrf-1* mutant hermaphrodites under both non-irradiated and UV-irradiated conditions following *cep-1* RNAi treatment (Fig 7D). In *rrf-1* mutants, although the germ line is susceptible to RNAi as in N2, many, but not all, somatic tissues are resistant to RNAi [61]. We found that RNAi depletion of *cep-1* dramatically repressed the transcriptional activation of *atg-18* gene following UV irradiation in *rrf-1* mutant hermaphrodites (Fig 7D). This result indicates that CEP-1 functions either in the germ line or in some specific somatic tissues, which are susceptible to RNAi in *rrf-1* mutants, for the transcriptional activation of autophagy genes. In summary, these results indicate that CEP-1 is required for induction of autophagy upon DNA damage and that CEP-1 activates some of the autophagy genes at the transcriptional level.

## Discussion

PGL-1 and PGL-3 are the major constitutive components of *C*. *elegans* germ granules termed P granules. In this study, we revealed that autophagy removes PGL-1 and PGL-3 from germ cells in adult hermaphrodite gonads upon UV-induced DNA damage. Why does autophagy remove PGL-1 and PGL-3 from germ cells upon DNA damage? Although it was previously shown that autophagy also eliminates PGL-1 and PGL-3 when they are missegregated to somatic blastomeres during embryogenesis [48], it has not been clearly answered why autophagy needs to specifically target PGL-1 and PGL-3. It was demonstrated through the study of *mes-1* mutants that P granules do not necessarily alter or deteriorate preprogrammed somatic development when they are missegregated to somatic tissues [62]. In this sense, although autophagic elimination of PGL-1 and PGL-3 in somatic cells may serve as a fail-safe mechanism, in fact, autophagy does not have to remove PGL-1 and PGL-3 from somatic blastomeres to assure or protect normal somatic development. In previous studies, we found that PGL-1 and PGL-3 serve as critical repressors of apoptosis not only in the germ line but also in the soma by repressing both the protein level of CED-4 and the cytoplasmic translocation of SIR-2.1 [31, 63]. From our previous and current studies, we propose that autophagy specifically targets PGL-1 and PGL-3 primarily to synergistically link autophagy to apoptosis during postembryonic germline development, that is, to induce a higher level of germ cell apoptosis following DNA damage. Whereas autophagy functions to degrade organelles within cells, apoptosis functions to eliminate cells within organisms. Depending on the circumstances, autophagy and apoptosis act either cooperatively or competitively [64]. In many cases, autophagy blocks the induction of apoptosis, while activation of apoptosis-associated caspase shuts off the process of autophagy [65]. However, consistent with our results, autophagy can also help to induce apoptosis. Several studies have demonstrated that autophagy is required to activate apoptosis under stress conditions [66–68]. In this study, we identified PGL-1 and PGL-3 as the key molecules that link autophagy to apoptosis during DNA damage-induced germ cell apoptosis. A synergistic link between autophagy and apoptosis has also been observed during *Drosophila* oogenesis, in which autophagic degradation of dBruce, an inhibitor of apoptosis, facilitates apoptosis of nurse cells [69]. It will be interesting to examine whether a similar synergistic link between autophagy and apoptosis also exists during mammalian oogenesis and/or spermatogenesis to maintain the integrity and quality of the gametes.

It appears that loss of PGL-1 occurs very early during *C*. *elegans* germ cell apoptosis under both physiological and DNA-damaged conditions likely as a decisive and irreversible event [31, 70]. We propose that monitoring removal of PGL-1 and/or PGL-3 from germ cells can serve as an alternative method for sensitive detection, time-lapse observation, and even quantification of apoptotic germ cells, with the caveat that scoring PGL-absent germ cells provides significantly larger numbers than previous apoptosis counting methods, because, compared to the previous methods, loss of PGL-1 and PGL-3 occurs very early during the apoptosis and stably remains until the apoptotic germ cells are finally engulfed and consumed [31].

We have shown that the removal of PGL-1 and PGL-3 from germ cells in adult hermaphrodite gonads following DNA damage requires largely the same set of autophagy genes as the ones required for the removal of PGL-1 and PGL-3 that are missegregated to somatic blastomeres during embryogenesis. However, our data also suggest that the removal of PGL proteins from gonadal germ cells requires a different mechanism for substrate recognition as compared to the one used in somatic blastomeres. Further studies are required to determine the exact mechanism of P granule targeting in gonadal germ cells for the autophagic degradation.

Our data are consistent with the hypothesis that the removal of PGL proteins by autophagy is required for the full induction of germ cell apoptosis upon DNA damage. Multiple lines of evidence support this model. A previous report had indicated that DNA damage-induced germ cell apoptosis depends on autophagy [54]. Our previous data had demonstrated that P granules are massively degraded in apoptotic germ cells upon DNA damage [31]. We now show that autophagy induction precedes the P granule degradation and that autophagy, as evidenced by LGG-1 foci accumulation, closely associates with and depletes the major P granule component, PGL-1. Our model is supported by the genetic interactions we have uncovered. The apoptosis defect of autophagy mutants is bypassed by depletion of *pgl-1* or *pgl-3*. We have also revealed that a number of autophagy genes are transcriptionally activated following DNA damage and that this activation requires CEP-1, the worm p53-like protein needed for induction of germ cell apoptosis upon DNA damage.

Autophagy, as measured by LGG-1 foci formation, was almost absent without DNA damage, but LGG-1 foci accumulated soon after UV irradiation in the pachytene-stage germ cells in wild-type adult hermaphrodite gonads. The finding that LGG-1 foci formation upon DNA damage was significantly reduced in *pgl-1* and *pgl-3* mutant hermaphrodite gonads suggests that PGL-1 and PGL-3 are possibly the major substrates of autophagy, because PGL-3, which itself is the substrate of autophagy, is required for the autophagic degradation of PGL-1 in somatic blastomeres [48]. The view that PGL-1 and PGL-3 serve as the substrates of autophagy in adult hermaphrodites was also supported by the results of our western blot analysis showing that the protein level of PGL-1 was reduced following UV irradiation in adult hermaphrodites in an autophagy activity-dependent manner. In contrast, LGG-1 foci persisted with or without UV irradiation in *glh-1* mutant hermaphrodite gonads, in which PGL-1 and PGL-3 are dissociated from the perinuclear region and dispersed throughout the cytoplasm [26, 27, 59]. These results suggest that not only the presence of PGL-1 and PGL-3 but also their subcellular localization, that is, their dispersal to the cytoplasm is important for the activation of autophagy. We hypothesize that, although autophagy is not activated when PGL-1 and PGL-3 are normally localized to the perinuclear region, autophagy is activated when PGL-1 and PGL-3 are dispersed to the cytoplasm, as exemplified in somatic blastomeres during embryogenesis. In these cells, missegregated PGL-1 and PGL-3 are dispersed to the entire cytoplasm and autophagy is activated without DNA damage [48]. The coincidence between dispersal of PGL-1 and PGL-3 to the entire cytoplasm and persistent formation of LGG-1 foci is also observed in developing oocytes and newly fertilized embryos [26, 41]. It remains to be determined whether subcellular localization or physiological status of PGL-1 and/or PGL-3 is indeed affected upon DNA damage in the pachytene-stage germ cells for the activation of autophagy.

The p53 family of tumor suppressors trigger autophagy in mammalian cancer cells in response to genotoxic and/or environmental stimuli, mediated by the nutrient energy sensor AMP-activated protein kinase (AMPK), by inhibition of the mammalian target of rapamycin (mTOR), and by induction of the autophagy modulator DRAM1 [71, 72]. Furthermore, it was recently described that p53 transcriptionally activates the expression of Sestrins, the highly conserved stress-responsive proteins that promote AMPK signaling for the formation of autophagic vesicles [73]. These results indicate that p53 activates autophagy through several mediators. In *C*. *elegans*, CEP-1 has been shown to play critical roles during DNA damage responses in the germ line. When DNA damage induces a higher level of germ cell apoptosis in *C*. *elegans* adult hermaphrodites, the DNA damage signal is transduced through multiple gene products in the DNA damage checkpoint pathway to activate CEP-1 [13, 74]. Remarkably, *cep-1* mutants show no increase in germ cell apoptosis upon DNA damage [13]. CEP-1 has been shown to induce transcription of both *egl-1* and *ced-13* that encode two pro-apoptotic BH3-only proteins in response to DNA damage to increase germ cell apoptosis [14]. However, although the increase in germ cell apoptosis following DNA damage was reduced, the increase was not completely abrogated in *egl-1* single, *ced-13* single, and even *egl-1; ced-13* double mutants [9, 14]. These results suggest the presence of additional transcriptional targets of CEP-1 for full induction of germ cell apoptosis following DNA damage. In this study, we found that not only the formation of LGG-1 foci, but also the transcription of several autophagy genes, was induced by CEP-1 following DNA damage in adult hermaphrodites. These results suggest that autophagy genes are previously unidentified transcriptional targets of CEP-1 to increase germ cell apoptosis following DNA damage.

Germ granules, the germline-specific cytoplasmic structures, are also observed in various mammals. Germ granules have been implicated in the formation or maintenance of the germ line. As in *C*. *elegans*, the vast majority of mammalian germ cells undergo apoptosis. Germ cell apoptosis is critical to maintain the quality of germ line because germ cell apoptosis eliminates damaged or compromised germ cells from being used for the next generation. Using the *C*. *elegans* germ line, which serves as a prime model for mammalian germ cell apoptosis, we implicated the autophagic removal of P granules, the *C*. *elegans* germ granules, in the induction of germ cell apoptosis upon DNA damage. It will be interesting to further follow up on the mechanistic details and to analyze if removal of germ granules also has an apoptotic role in mammalian germ lines.

## Materials and methods

### *C*. *elegans* genetics

All strains were maintained at 20°C on nematode growth medium (NGM) agar plates seeded with *Escherichia coli* OP50 as previously described [75]. The strains used in this study are listed in S1 Table.

### UV irradiation

As a source of DNA damage, we solely used UV irradiation in this study. L4-stage hermaphrodites were pre-cultured for 24 h at 20°C, irradiated with 400 J/m^2^ of UV-C light (Sankyo Denki germicidal lamp G40T10, 40W, 254 nm) on OP50-seeded NGM agar plates, post-cultured on the plates for either 3 h (for observation of LGG-1 foci formation) or 24 h (for apoptotic germ cell counting) at 20°C, and subjected to respective experiments.

### Immunofluorescence analysis and LGG-1 foci counting

Immunofluorescence analysis was performed as previously described [31]. In brief, worms were dissected to extrude gonads in 10 μl of M9 buffer containing 100 μg/ml tetramisole on a poly-L-lysine-coated microscope slide, covered with a coverslip, freeze-cracked with liquid nitrogen, fixed with cold methanol and cold acetone, and immunostained with primary and secondary antibodies. The specimens were further counterstained with 1 μM TO-PRO-3 (Molecular Probes) to stain DNA, and observed under a confocal microscope (Olympus, FV1000 Spectral). The following primary antibodies were used: mouse monoclonal OIC1D4, which specifically recognizes PGL-1 (undiluted; a kind gift from Susan Strome), rabbit anti-PGL-3 (1:400; a kind gift from Asako Sugimoto), rabbit anti-LGG-1 (1:400; a kind gift from Ken Sato), rabbit anti-GFP (1:400; Novus). The number of LGG-1 foci in immunofluorescent gonad images was scored using NIS-Elements 3.1 software (Nikon Instruments). To do this, we used the “Object Count” function of the software to identify immunostained LGG-1 foci in the pachytene region of the gonads by setting a threshold to distinguish LGG-1 foci from backgrounds. Thresholds were set arbitrarily for respective immunofluorescence images, but when a series of immunofluorescence images were analyzed for quantitative comparison, the threshold was set constant. More than 10 immunofluorescent gonad images were examined to determine the number and distribution of LGG-1 foci in respective genetic backgrounds under respective conditions.

### Western blot analysis

Western blot analysis was performed using whole worm protein extract obtained from ca. 100 gravid adult hermaphrodites of each genotype under each condition per gel well. Antibodies bound to a nitrocellulose membrane (PROTRAN BA83, Whatman) were visualized with ECL western blotting detection kit (Amersham), and respective band intensities were measured with LAS-3000 image analyzer using Multi Gauge (v.3.0) software (Fuji Film). To quantify band intensity, we used the “ROI” tool to define the band area, and employed the “Analyze” tool to measure the intensity of respective protein bands on a western blot image. Protein band intensity was presented as a sum of optical density in the ROI using an arbitrary unit. Then, the intensity of each PGL-1 protein band was normalized with that of α-tubulin band on the same lane. Finally, the normalized PGL-1 band intensities were converted to “relative values”, so as to make the PGL-1 band intensities under non-irradiated (0 J/m^2^ UV) condition on respective western blot images as value 1. Then, obtained “relative values” (3 values for each genetic background under each condition) were plotted, averaged, and statistically evaluated using *t*-test. The following primary and secondary antibodies were used: rabbit anti-PGL-1 (1:4000) [26], mouse anti-α-tubulin (1:2000; Sigma), HRP-conjugated goat anti-rabbit IgG (1:10000; Santa Cruz Biotech.), and HRP-conjugated donkey anti-mouse IgG (1:1000; Jackson ImmunoResearch).

### RNA interference (RNAi)

RNAi experiments were performed using “soaking” method as described previously [76]. Briefly, L1-stage worms soaked in respective dsRNA solutions for 24 h were recovered to OP50-seeded NGM agar plates, grown for a few days until they reached the young adult stage (24 h after the L4 stage), irradiated or not irradiated with UV, further incubated for 24 h, and they (P0) or their progeny (F1) were examined for the resulting RNAi phenotype.

### Germline apoptosis assay

Apoptotic germ cells were visualized by Acridine Orange (AO) vital staining as previously described [55], with minor modifications. Briefly, UV-irradiated or non-irradiated worms were stained with 25 μg/ml of Acridine Orange (AO) in M9 buffer for 1 h in the dark, allowed to recover on fresh OP50-seeded NGM plates for 20 min, and observed under fluorescence microscopy to count the number of Acridine Orange (AO)-positive germ cells per gonad arm. Only one gonad arm was scored for each observed animal. 30–40 animals were examined for each experiment.

### Real time RT-PCR (qRT-PCR)

Adult hermaphrodites of respective genotypes, which were treated or not treated with UV irradiation, were collected in TRIzol (Invitrogen), and total RNA was extracted using a phase lock gel (MaXtract High Density, Qiagen). cDNA was synthesized using oligo-dT primer and M-MLV reverse transcriptase (Invitrogen). qPCR reactions were performed using *Power* SYBR Green PCR Master Mix (Applied Biosystems). The final PCR volume was 25 μl. *act-1* mRNA was used as an endogenous control for data normalization. The primers used in this study are listed in S2 Table.

### Statistical analysis

All experiments were repeated more than three times, and *p*-values were calculated using either Student’s *t*-test or one-way ANOVA test for statistical evaluation of data.

## Supporting information

S1 Table List of *C. elegans* strains used in this study. (DOCX)

S2 Table List of qPCR primers used in this study. (DOCX)

S3 Table Numerical datasets that underlie respective graphs. (XLSX)

S4 Table Statistical analysis of Fig 1 and Fig 2 data by one-way ANOVA. (DOCX)

S1 Fig LGG-1 foci formation in autophagy mutant hermaphrodite gonads with or without UV irradiation. (A) Hermaphrodites of wild-type N2, *atg-13(bp414)*, *atg-9(bp564)*, *epg-8(bp251)*, *atg-3(bp412)*, *atg-4*.*1(bp501)*, *atg-4*.*2(tm3948)*, *atg-2(bp576)*, and *atg-18(gk378)* were irradiated or not irradiated with 400 J/m^2^ of UV at 24 h post the L4 stage, collected at 3 h after the UV irradiation, and dissected and immunostained with anti-LGG-1 antibody (green) along with DNA counterstaining (blue). Pachytene region of their gonads is shown. d, distal side of each gonad arm. Scale bar, 20 μm. (B) The four distinct steps of autophagic process and autophagy genes examined in this study, which function in respective steps. (C) Box-and-whisker plots depicting the number of LGG-1 foci formed in the pachytene region of hermaphrodite gonad arms in N2 and respective autophagy mutants with or without 400 J/m^2^ of UV irradiation. Horizontal lines in respective boxes represent the median. Upper lines and lower lines extended from respective boxes represent 75% quartile and 25% quartile, respectively. Gray dots indicate numbers of LGG-1 foci formed in the pachytene region of respective gonad arms. Number of analyzed gonads, n ≥ 10 for all the strains in respective conditions. Statistical significance was calculated using Student’s *t*-test. ***, *p* < 0.001 against UV-irradiated N2 gonads.
(PDF)

S2 Fig Localization of PGL-1 and PGL-3 in germ cells in wild-type N2 hermaphrodite gonads under physiological and DNA-damaged conditions. Late-pachytene region of wild-type N2 adult hermaphrodite gonads, which were irradiated (400 J/m^2^) or not irradiated (0 J/m^2^) with UV, dissected, fixed, and immunostained with both anti-PGL-1 (red) and anti-PGL-3 (green) antibodies along with TO-PRO-3 DNA staining (blue). Merged images between PGL-1 (red) and DNA (blue) signals and between PGL-3 (green) and DNA (blue) signals are also shown. d, distal side of each gonad arm. Scale bar, 20 μm.
(PDF)

S3 Fig Time-lapse live imaging of *Ppie-1::GFP::lgg-1* expression in a hermaphrodite gonad following UV irradiation. (A) Hermaphrodites carrying an integrated *Ppie-1*::*GFP*::*lgg-1* transgene in *asp-10(tm6801)* genetic background were treated with *vha-5* and *vha-13* double RNAi depletion at the L1 larval stage to suppress quick turnover of LGG-1 foci by reducing the activities of lysosomal enzymes [43]. Then, these hermaphrodites were, or were not, treated with 400 J/m^2^ of UV irradiation at 24 h post the L4 stage, immediately mounted on agar pad with a drop of M9 buffer containing 0.2 mM tetramisole on a microscope slide, covered with a coverslip, the edges of which were sealed with melted Valap to avoid drying of the specimen [77]. Finally, the gonads of mounted live hermaphrodites were periodically imaged under a confocal fluorescence microscope at 0.5 h, 1.5 h, 3 h, and 4.5 h after the UV irradiation. d, distal side of each gonad arm. Scale bar, 20 μm. (B) Enlarged images of insets (the areas enclosed with white dotted squares) in (A), which correspond to the late pachytene region of respective gonads, at 1.5 h and 3 h after the UV irradiation. (C) Mean ± s.d. number of LGG-1 foci formed in the pachytene region of *Ppie-1*::*GFP*::*lgg-1* transgenic hermaphrodite gonads at respective time points following 0 J/m^2^ (white bars) or 400 J/m^2^ (black bars) of UV irradiation. Number of gonads observed up to 4.5 h following UV irradiation for time-lapse live imaging, n = 9 for respective conditions.
(PDF)

S4 Fig Our *sepa-1* RNAi treatment effectively suppressed ectopic formation of PGL granules in somatic blastomeres in autophagy mutant embryos. Autophagy mutants, *atg-3(bp412)*, *atg-4*.*1(bp501)*, *atg-7(bp411)*, and *atg-18(gk378)*, were treated or not treated with *sepa-1* (M01E5.6) RNAi depletion in their P0 generation, and their F1 embryos were fixed and immunostained with anti-PGL-1 antibody (green) along with TO-PRO-3 DNA staining (blue). Note that the two blastomeres, which were immunostained strongly and consistently with anti-PGL-1 antibody with or without *sepa-1* RNAi, are Z2 and Z3 embryonic germline precursor cells and not somatic blastomeres. Scale bar, 20 μm. Number of embryos examined, n ≥ 10 for respective autophagy mutants after respective RNAi treatments.
(PDF)

S5 Fig SEPA-1::GFP was not expressed in germ cells of adult hermaphrodite gonads. (A) A fluorescence image of an intact *Psepa-1*::*sepa-1*::*GFP* transgenic adult hermaphrodite. (B) A fluorescence image of a dissected *Psepa-1*::*sepa-1*::*GFP* transgenic adult hermaphrodite. (C) A Nomarski DIC image of (B). SEPA-1::GFP expression was observed in the anterior and posterior portions of the intestine (yellow arrowheads) and in the embryos (red arrowheads), but not in the germ cells of their gonads. h, head of the animal. d, distal end of the gonad. Scale bars, 100 μm. Number of worms examined, n = 7.
(PDF)

S6 Fig The formation of LGG-1 foci following UV irradiation was reduced in *vet-6* mutant hermaphrodite gonads. (A) N2 and *vet-6(tm1226)* hermaphrodites were irradiated or not irradiated with 400 J/m^2^ of UV at 24 h post the L4 stage, collected at 3 h after the UV irradiation, and dissected and immunostained with anti-LGG-1 antibody (green) along with DNA counterstaining (blue). Pachytene region of their gonads is shown. d, distal side of each gonad arm. Scale bar, 20 μm. (B) Box-and-whisker plots depicting the number of LGG-1 foci formed in the pachytene region of N2 and *vet-6(tm1226)* hermaphrodite gonads with or without 400 J/m^2^ of UV irradiation. The box-and-whisker plots are drawn as in S1C Fig. Number of analyzed gonads, n ≥ 10 for both of the strains in respective conditions. Statistical significance was calculated using Student’s *t*-test. ***, *p* < 0.001.
(PDF)

S7 Fig The formation of LGG-1 foci in N2, *cep-1*, and *egl-1* mutant hermaphrodite gonads without UV irradiation. (A) Wild-type N2, *cep-1(gk138)*, and *egl-1(n487)* hermaphrodites were dissected and immunostained with anti-LGG-1 antibody (green) along with DNA counterstaining (blue). Pachytene region of their gonads is shown. d, distal side of each gonad arm. Scale bar, 20 μm. (B) Box-and-whisker plots depicting the number of LGG-1 foci formed in the pachytene region of gonad arms from N2, *cep-1(gk138)*, and *egl-1(n487)* adult hermaphrodites without UV irradiation. The box-and-whisker plots are drawn as in S1C Fig. Number of analyzed gonads, n ≥ 10 for respective strains. Statistical significance was calculated using Student’s *t*-test. n.s., *p* > 0.05 against N2 gonads.
(PDF)
