# Supplementary material for: Autophagy of germ-granule components, PGL-1 and PGL-3, contributes to DNA damage-induced germ cell apoptosis in C. elegans
Source: PLoS Genet. 2019 May 24;15(5):e1008150. doi: 10.1371/journal.pgen.1008150 (PMC6534287; doi:10.1371/journal.pgen.1008150)
Supplement: S1 Table — (DOCX) [file pgen.1008150.s001.docx]

**Table S1. List of *C. elegans* strains used in this study**

| **Strain** | **Genotype** |
| --- | --- |
| N2 | *C. elegans* wild isolate |
| HZ1683 | *him-5(e1490) V; atg-2(bp576) X* |
| HZ1684 | *atg-3(bp412) IV; him-5(e1490) V* |
| HZ1685 | *atg-4.1(bp501) I* |
| FX03948 | *atg-4.2(tm3948) IV* |
| HZ1686 | *bnIs1[pie-1p::GFP::pgl-1 + unc-119(+)] I; atg-7(bp411) IV; him-5(e1490) V* |
| HZ1687 | *atg-9(bp564) him-5(e1490) V* |
| HZ1688 | *atg-13(bp414) III* |
| VC893 | *atg-18(gk378) V* |
| FX3425 | *epg-5(tm3425) II* |
| HZ1691 | *epg-8(bp251) I; him-5(e1490) V* |
| HZ1692 | *epg-9(bp320) IV; him-5(e1490) V* |
| HZ455 | *him-5(e1490) V; bpIs131[Psepa-1::sepa-1::GFP+unc-76(+)]* |
| SS519 | *pgl-1(ct131) him-3(e1147) IV* |
| SS608 | *pgl-3(bn104) V* |
| SS618 | *pgl-1(ct131) him-3(e1147) IV; pgl-3(bn104) V* |
| RB656 | *glh-1(ok439) I* |
| YHS136 | *pgl-1(ct131) him-3(e1147) IV; atg-18(gk378) V* |
| YHS137 | *epg-5(tm3425) II; pgl-1(ct131) him-3(e1147) IV* |
| YHS147 | *epg-5(tm3425) II; pgl-3(bn104) V* |
| GK682 | *dkIs398[Ppie-1::gfp::lgg-1, unc-119(+)] I; unc-119(ed3) III* |
| YHS201 | *dkIs398[Ppie-1::gfp::lgg-1, unc-119(+)] I; sam37[pgl-1::mTagRFPT::3XFLAG] IV; asp-10(tm6801)* *V* |
| YHS203 | *dkIs398[Ppie-1::gfp::lgg-1, unc-119(+)] I; unc-119(ed3) III; him-8(e1489) IV* |
| TJ1 | *cep-1(gk138) I* |
| MT1082 | *egl-1(n487) V* |
| RB1293 | *vet-2(ok1392) I* |
| FX01226 | *vet-6(tm1226) I* |
| FX10601 | *zk1053.3(tm10601) I* |
| NL2098 | *rrf-1(pk1417) I* |

YHS strains were made for this study by us.
