# Supplementary material for: Autophagy of germ-granule components, PGL-1 and PGL-3, contributes to DNA damage-induced germ cell apoptosis in C. elegans
Source: PLoS Genet. 2019 May 24;15(5):e1008150. doi: 10.1371/journal.pgen.1008150 (PMC6534287; doi:10.1371/journal.pgen.1008150)
Supplement: S2 Table — (DOCX) [file pgen.1008150.s002.docx]

**Table S2. List of qPCR primers used in this study**

| **To check mRNA levels of each gene** | |
| --- | --- |
| *act-1*_qPCR_F | 5’-CCAGGAATTGCTGATCGTATGCAGAA-3’ |
| *act-1*_qPCR_R | 5’-TGGAGAGGGAAGCGAGGATAGA-3’ |
| *atg-4.1*_qPCR_F | 5’- CATTGACATTTGAGCCGCCT-3’ |
| *atg-4.1*_qPCR_R | 5’- CATTGCCTCGATTCCATCTTCC-3’ |
| *atg-9*_qPCR_F | 5’- TTAATAAGCGAATCCTTCACCC-3’ |
| *atg-9*_qPCR_R | 5’- GCTCGTGATGTTTGTACTCCT-3’ |
| *atg-18_qPCR_F* | 5’- AGACTCTAAAGTTATATGCGTGGG-3’ |
| *atg-18_qPCR_R* | 5’- AGTTGTTGAGACCAAGATGTG-3’ |
| *lgg-1_qPCR_F* | 5’- GAGAACAACTTTGAGAAGCGT-3’ |
| *lgg-1_qPCR_R* | 5’- TCGTGATGGTCCTGGTAGAG-3’ |
