# Supplementary material for: Autophagy of germ-granule components, PGL-1 and PGL-3, contributes to DNA damage-induced germ cell apoptosis in C. elegans
Source: PLoS Genet. 2019 May 24;15(5):e1008150. doi: 10.1371/journal.pgen.1008150 (PMC6534287; doi:10.1371/journal.pgen.1008150)
Supplement: S4 Table — (DOCX) [file pgen.1008150.s004.docx]

**Table S4A. Statistical analysis of AO-positive germ cells in Figure 1B by one-way Anova**

| **Group** | **Count** | **Sum** | **Average** | **Variance** | ***p*-value** |
| --- | --- | --- | --- | --- | --- |
| N2 | 39 | 161 | 4.12821 | 1.21997 |  |
| ***atg-13*** | 33 | 262 | 7.93939 | 5.05871 | **6.252E-14** |
| ***atg-9*** | 31 | 183 | 5.90323 | 2.55699 | **6.625E-07** |
| *epg-8* | 42 | 167 | 3.97619 | 5.58479 | 0.7152171 |
| *atg-3* | 33 | 153 | 4.63636 | 2.05113 | 0.0938502 |
| ***atg-4.1*** | 36 | 241 | 6.69444 | 1.76111 | **1.069E-13** |
| ***atg-4.2*** | 27 | 205 | 7.59259 | 3.01994 | **1.526E-14** |
| *atg-2* | 25 | 108 | 4.32 | 2.39333 | 0.5649818 |
| *atg-18* | 38 | 139 | 3.65789 | 1.85277 | 0.0997034 |
| *epg-5* | 27 | 113 | 4.18519 | 1.00285 | 0.831276 |
| *epg-9* | 39 | 190 | 4.87159 | 5.16734 | 0.0700578 |

**Table S4B. Statistical analysis of AO-positive germ cells in Figure 1C by one-way Anova**

| **Group** | **Count** | **Sum** | **Average** | **Variance** | ***p*-value** |
| --- | --- | --- | --- | --- | --- |
| **N2-mock RNAi (UV-)** | 22 | 93 | 4.227273 | 2.088745 |  |
| **N2-mock RNAi (UV+)** | 26 | 188 | 7.230769 | 1.384615 | **3.65E-10** |
| N2-*cep-1* RNAi (UV-) | 21 | 83 | 3.952381 | 1.347619 |  |
| N2-*cep-1* RNAi (UV+) | 23 | 90 | 3.913043 | 1.083004 | 0.906211 |
| *atg-13*-mock RNAi (UV-) | 27 | 153 | 5.666667 | 1.230769 |  |
| *atg-13*-*cep-1* RNAi (UV-) | 27 | 157 | 5.814815 | 1.079772 | 0.614694 |
| *atg-9*-mock RNAi (UV-) | 27 | 151 | 5.592593 | 0.635328 |  |
| *atg-9*-*cep-1* RNAi (UV-) | 25 | 142 | 5.68 | 0.726667 | 0.703986 |
| *atg-4.1*-mock RNAi (UV-) | 22 | 162 | 7.363636 | 2.4329 |  |
| *atg-4.1*-*cep-1* RNAi (UV-) | 44 | 309 | 7.022727 | 7.139006 | 0.582895 |
| *atg-4.2*-mock RNAi (UV-) | 22 | 149 | 6.772727 | 3.517316 |  |
| *atg-4.2*-*cep-1* RNAi (UV-) | 50 | 320 | 6.4 | 3.183673 | 0.424141 |

**Table S4C. Statistical analysis of relative apoptosis levels in Figure 2 by one-way Anova**

| **Group** | **Count** | **Ratio** | **Variance** | ***p*-value** |
| --- | --- | --- | --- | --- |
| ***atg-13* UV(+/-)** | 25 | 0.90558 | 0.16736 |  |
| ***atg-13; pgl-1* RNAi UV(+/-)** | 46 | 1.50850 | 0.21377 | **0.007017** |
| ***atg-13* UV(+/-)** | 25 | 0.90558 | 0.16736 |  |
| ***atg-13; pgl-3* RNAi UV(+/-)** | 45 | 1.30919 | 0.28523 | **0.047744** |
| ***atg-9* UV(+/-)** | 20 | 1.13728 | 0.18865 |  |
| ***atg-9; pgl-1* RNAi UV(+/-)** | 39 | 1.36148 | 0.28532 | **0.013624** |
| ***atg-9* UV(+/-)** | 20 | 1.13728 | 0.18865 |  |
| ***atg-9; pgl-3* RNAi UV(+/-)** | 34 | 1.80447 | 0.21145 | **0.013282** |
| ***epg-8* UV(+/-)** | 20 | 0.77285 | 1.56842 |  |
| ***epg-8; pgl-1* RNAi UV(+/-)** | 42 | 1.36363 | 0.29154 | **0.003666** |
| ***epg-8* UV(+/-)** | 20 | 0.77285 | 1.56842 |  |
| ***epg-8; pgl-3* RNAi UV(+/-)** | 31 | 1.59540 | 0.21429 | **1.57E-05** |
| ***atg-3* UV(+/-)** | 23 | 0.85586 | 0.01999 |  |
| ***atg-3; pgl-1* RNAi UV(+/-)** | 52 | 1.45138 | 0.16736 | **0.004488** |
| ***atg-3* UV(+/-)** | 23 | 0.85586 | 0.01999 |  |
| ***atg-3; pgl-3* RNAi UV(+/-)** | 49 | 1.37350 | 0.29039 | **0.045684** |
| ***atg-4.1* UV(+/-)** | 24 | 1.09688 | 0.19408 |  |
| ***atg-4.1; pgl-1* RNAi UV(+/-)** | 62 | 1.34302 | 0.28865 | **0.000249** |
| ***atg-4.1* UV(+/-)** | 24 | 1.09688 | 0.19408 |  |
| ***atg-4.1; pgl-3* RNAi UV(+/-)** | 40 | 1.40937 | 0.27622 | **0.047549** |
| ***atg-4.2* UV(+/-)** | 23 | 0.94505 | 0.14343 |  |
| ***atg-4.2; pgl-1* RNAi UV(+/-)** | 40 | 1.63284 | 0.12603 | **0.03814** |
| ***atg-4.2* UV(+/-)** | 23 | 0.94505 | 0.14343 |  |
| ***atg-4.2; pgl-3* RNAi UV(+/-)** | 31 | 1.38698 | 0.23593 | **0.000577** |
| ***atg-2* UV(+/-)** | 23 | 0.90347 | 0.15101 |  |
| ***atg-2; pgl-1* RNAi UV(+/-)** | 25 | 1.82951 | 0.20099 | **2.29E-07** |
| ***atg-2* UV(+/-)** | 23 | 0.90347 | 0.15101 |  |
| ***atg-2; pgl-3* RNAi UV(+/-)** | 35 | 1.48447 | 0.22927 | **5.81E-05** |
| ***atg-18* UV(+/-)** | 23 | 0.95683 | 0.16158 |  |
| ***pgl-1; atg-18* UV(+/-)** | 51 | 1.25887 | 0.19408 | **3.06E-09** |
| ***atg-18* UV(+/-)** | 23 | 0.95683 | 0.16158 |  |
| ***atg-18; pgl-3* RNAi UV(+/-)** | 40 | 1.66091 | 0.21012 | **7.36E-11** |
| ***epg-5* UV(+/-)** | 23 | 1.03293 | 0.17101 |  |
| ***epg-5; pgl-1* UV(+/-)** | 55 | 1.32258 | 0.25362 | **3.21E-12** |
| ***epg-5* UV(+/-)** | 23 | 1.03293 | 0.17101 |  |
| ***epg-5; pgl-3* UV(+/-)** | 27 | 1.26984 | 0.29763 | **1.78E-06** |
| ***epg-9* UV(+/-)** | 23 | 0.81566 | 0.18088 |  |
| ***epg-9; pgl-1* RNAi UV(+/-)** | 50 | 1.42854 | 0.24343 | **0.005975** |
| ***epg-9* UV(+/-)** | 23 | 0.81566 | 0.18088 |  |
| ***epg-9; pgl-3* RNAi UV(+/-)** | 49 | 1.33803 | 0.25208 | **0.004495** |
