# Supplementary material for: Autophagy of germ-granule components, PGL-1 and PGL-3, contributes to DNA damage-induced germ cell apoptosis in C. elegans
Source: PLoS Genet. 2019 May 24;15(5):e1008150. doi: 10.1371/journal.pgen.1008150 (PMC6534287; doi:10.1371/journal.pgen.1008150)

A

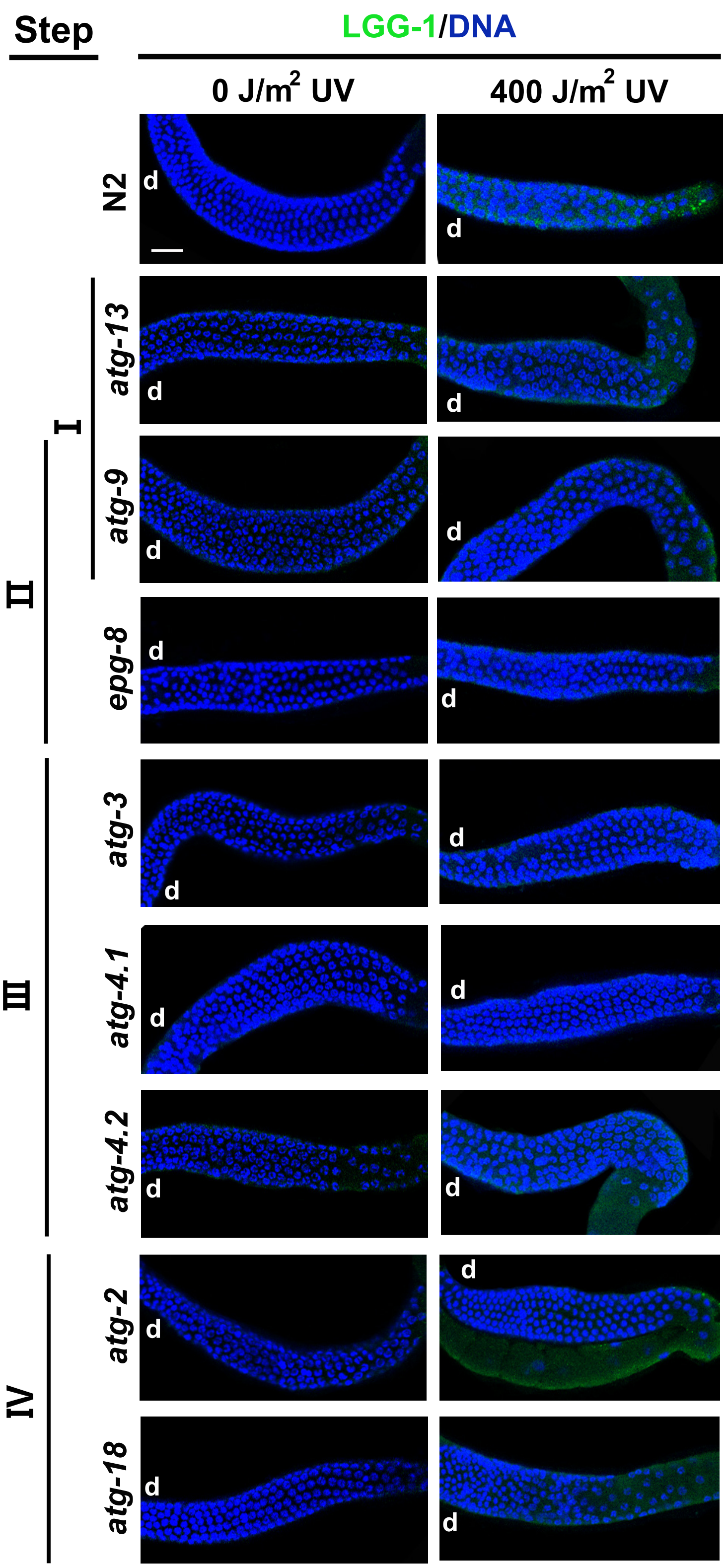

B

The steps of autophagy and involved genes

Ⅰ (induction) - *atg-13*, *atg-9*

Ⅱ (nucleation) - *atg-9*, *epg-8*

Ⅲ (elongation) - *atg-3*, *atg-4.1*, *atg-4.2*

Ⅳ (retrieval) - *atg-2*, *atg-18*

C

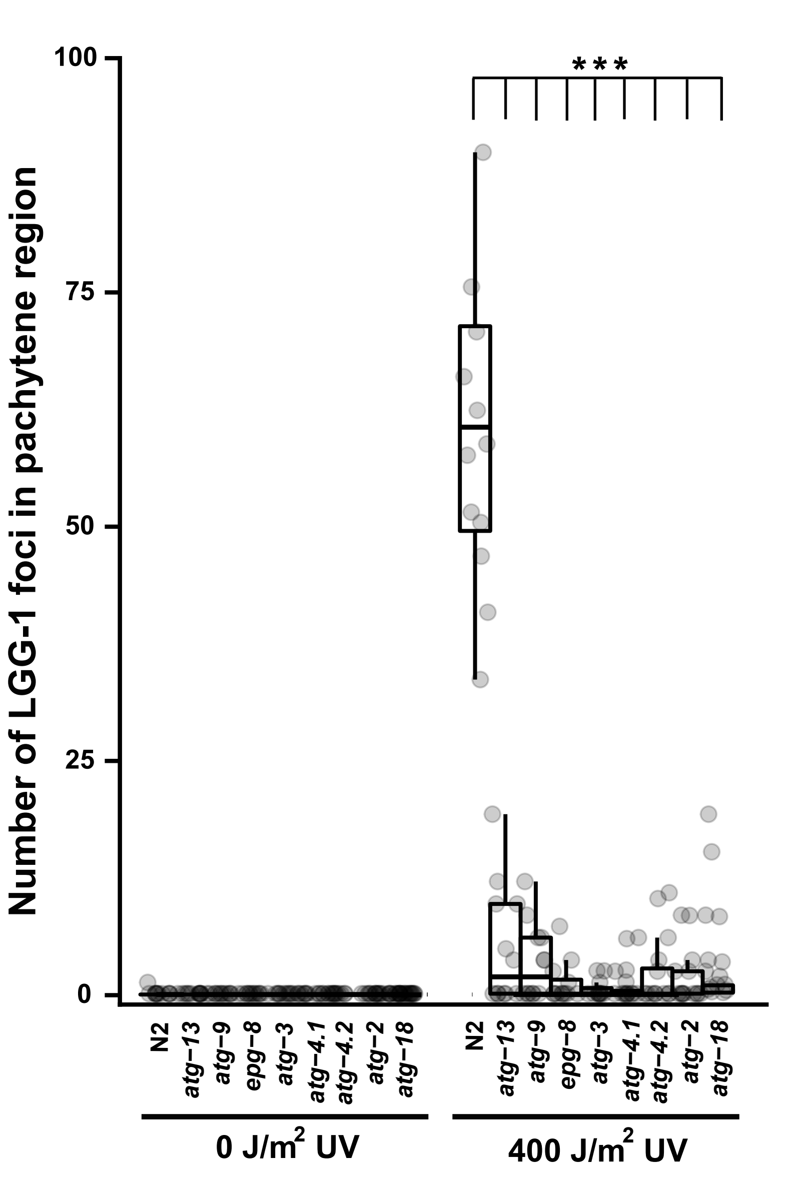

Supplement: S1 Fig — (A) Hermaphrodites of wild-type N2, atg-13(bp414), atg-9(bp564), epg-8(bp251), atg-3(bp412), atg-4.1(bp501), atg-4.2(tm3948), atg-2(bp576), and atg-18(gk378) were irradiated or not irradiated with 400 J/m2 of UV at 24 h post the L4 stage, collected at 3 h after the UV irradiation, and dissected and immunostained with anti-LGG-1 antibody (green) along with DNA counterstaining (blue). Pachytene region of their gonads is shown. d, distal side of each gonad arm. Scale bar, 20 μm. (B) The four distinct steps of autophagic process and autophagy genes examined in this study, which function in respective steps. (C) Box-and-whisker plots depicting the number of LGG-1 foci formed in the pachytene region of hermaphrodite gonad arms in N2 and respective autophagy mutants with or without 400 J/m2 of UV irradiation. Horizontal lines in respective boxes represent the median. Upper lines and lower lines extended from respective boxes represent 75% quartile and 25% quartile, respectively. Gray dots indicate numbers of LGG-1 foci formed in the pachytene region of respective gonad arms. Number of analyzed gonads, n ≥ 10 for all the strains in respective conditions. Statistical significance was calculated using Student’s t-test. ***, p < 0.001 against UV-irradiated N2 gonads. (PDF) [file pgen.1008150.s005.pdf]
