# Supplementary figures and images for: Autophagy of germ-granule components, PGL-1 and PGL-3, contributes to DNA damage-induced germ cell apoptosis in C. elegans
Source: PLoS Genet. 2019 May 24;15(5):e1008150. doi: 10.1371/journal.pgen.1008150 (PMC6534287; doi:10.1371/journal.pgen.1008150)

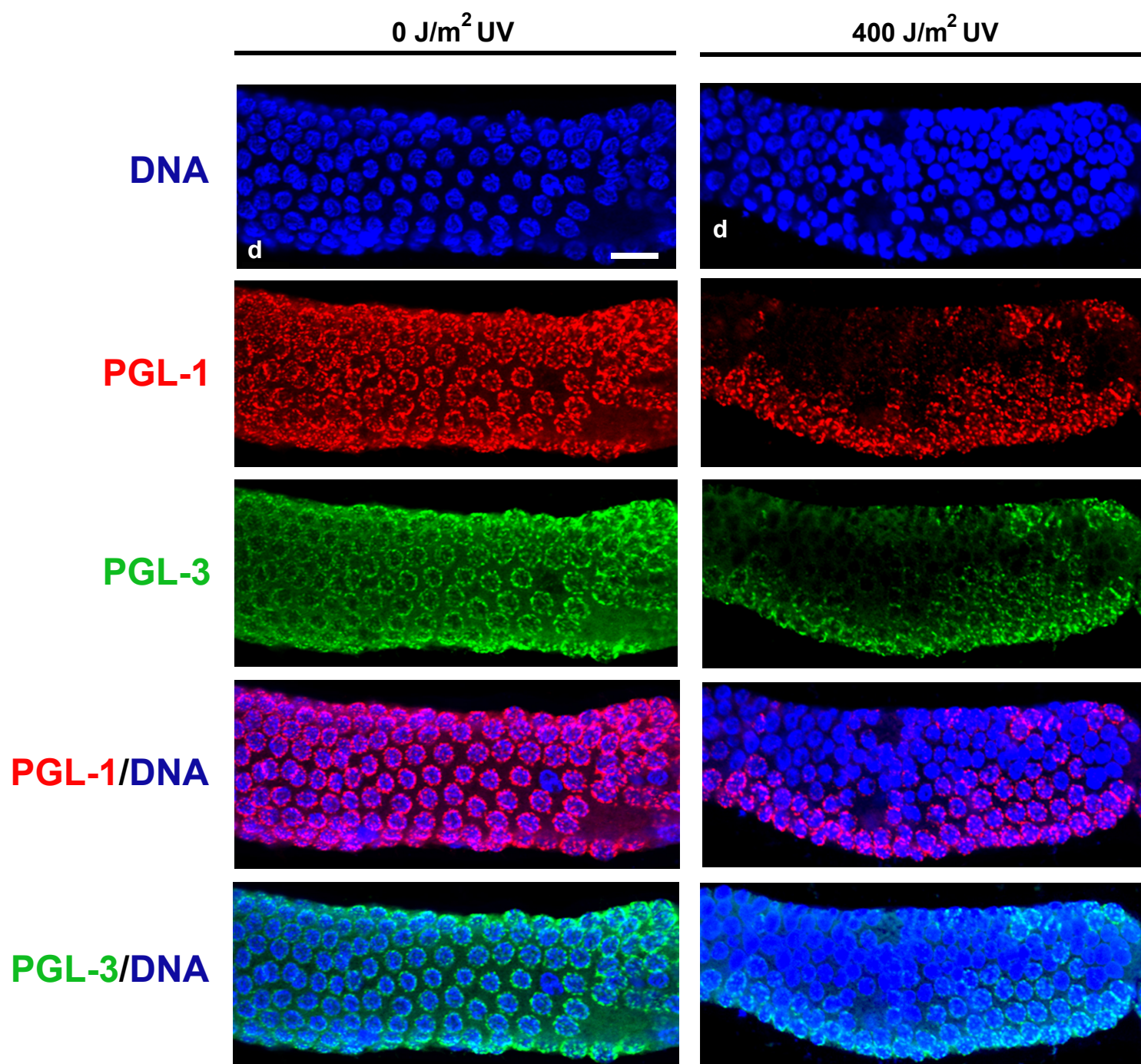

Supplement: S2 Fig — Late-pachytene region of wild-type N2 adult hermaphrodite gonads, which were irradiated (400 J/m2) or not irradiated (0 J/m2) with UV, dissected, fixed, and immunostained with both anti-PGL-1 (red) and anti-PGL-3 (green) antibodies along with TO-PRO-3 DNA staining (blue). Merged images between PGL-1 (red) and DNA (blue) signals and between PGL-3 (green) and DNA (blue) signals are also shown. d, distal side of each gonad arm. Scale bar, 20 μm. (PDF) [file pgen.1008150.s006.pdf]

**A***Ppie-1::GFP::lgg-1; asp-10(tm6801) + vha-5 and vha-13 RNAi*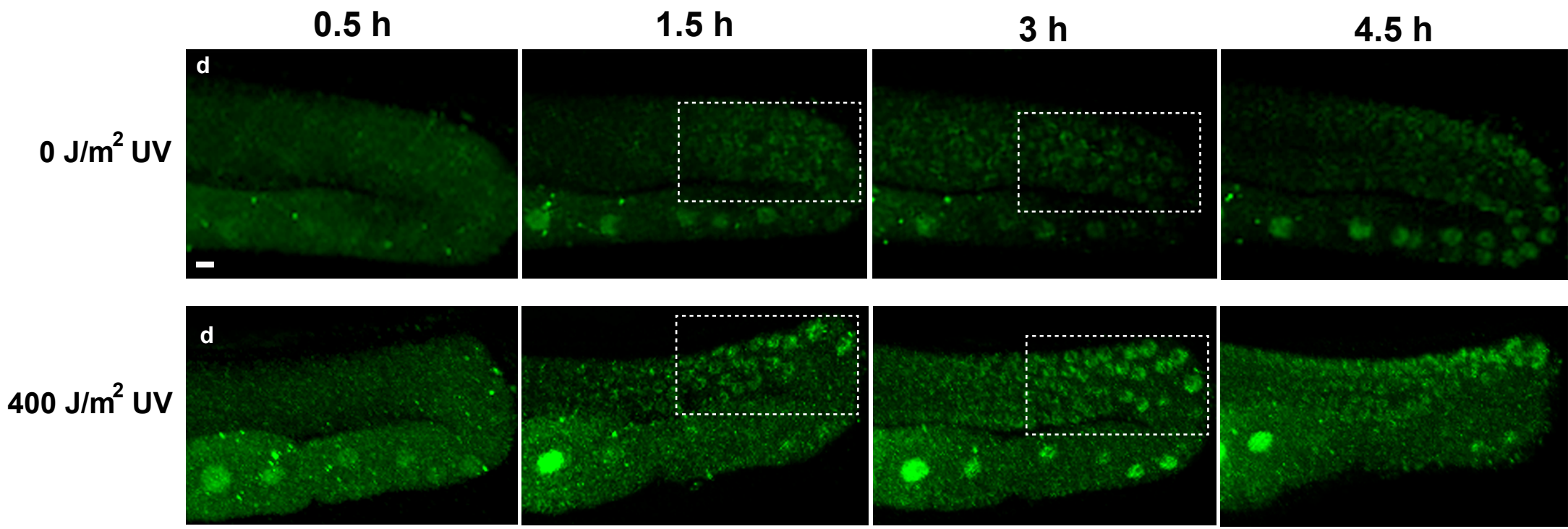**B**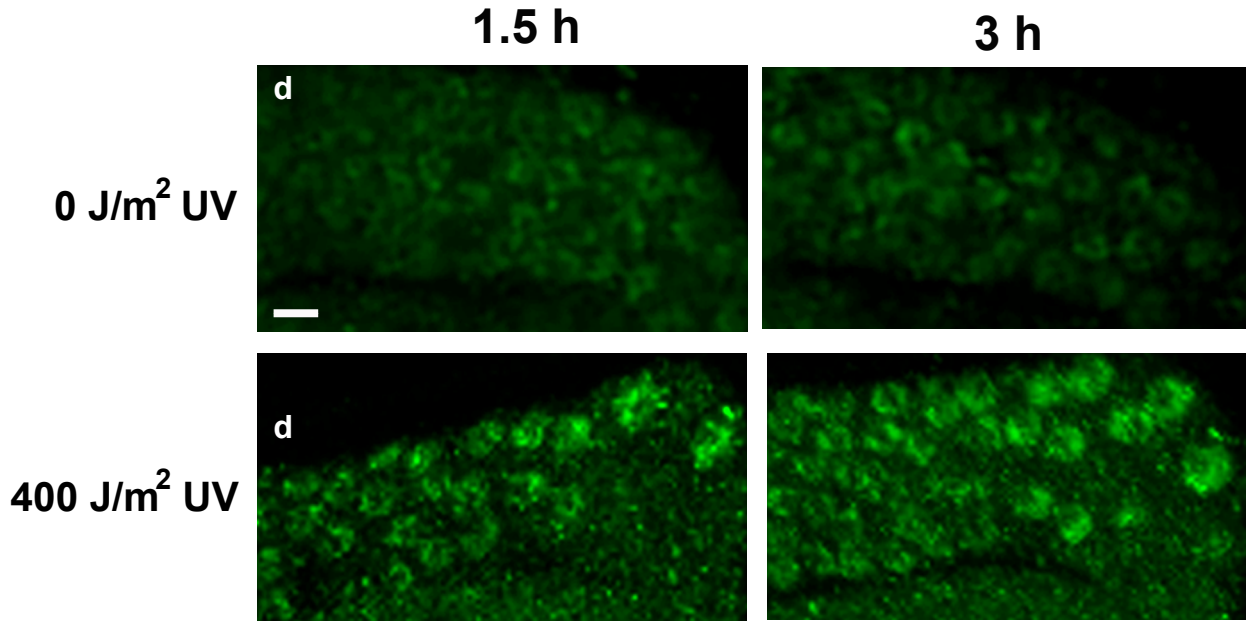**C**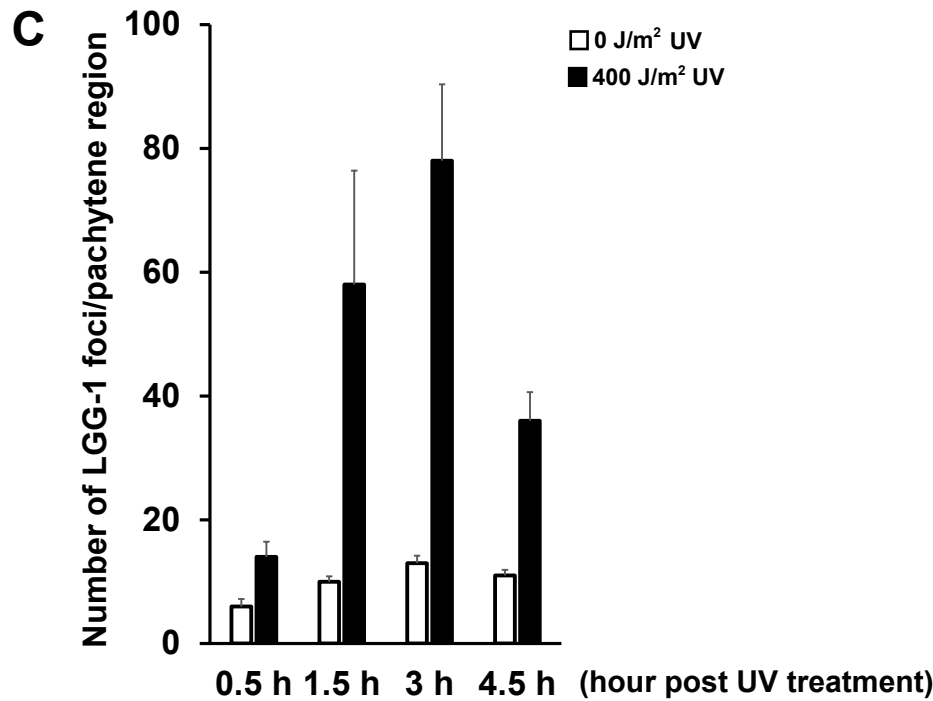

Supplement: S3 Fig — (A) Hermaphrodites carrying an integrated Ppie-1::GFP::lgg-1 transgene in asp-10(tm6801) genetic background were treated with vha-5 and vha-13 double RNAi depletion at the L1 larval stage to suppress quick turnover of LGG-1 foci by reducing the activities of lysosomal enzymes [43]. Then, these hermaphrodites were, or were not, treated with 400 J/m2 of UV irradiation at 24 h post the L4 stage, immediately mounted on agar pad with a drop of M9 buffer containing 0.2 mM tetramisole on a microscope slide, covered with a coverslip, the edges of which were sealed with melted Valap to avoid drying of the specimen [77]. Finally, the gonads of mounted live hermaphrodites were periodically imaged under a confocal fluorescence microscope at 0.5 h, 1.5 h, 3 h, and 4.5 h after the UV irradiation. d, distal side of each gonad arm. Scale bar, 20 μm. (B) Enlarged images of insets (the areas enclosed with white dotted squares) in (A), which correspond to the late pachytene region of respective gonads, at 1.5 h and 3 h after the UV irradiation. (C) Mean ± s.d. number of LGG-1 foci formed in the pachytene region of Ppie-1::GFP::lgg-1 transgenic hermaphrodite gonads at respective time points following 0 J/m2 (white bars) or 400 J/m2 (black bars) of UV irradiation. Number of gonads observed up to 4.5 h following UV irradiation for time-lapse live imaging, n = 9 for respective conditions. (PDF) [file pgen.1008150.s007.pdf]

PGL-1/DNA

RNAi treatment

mock

*sepa-1*

*atg-3*

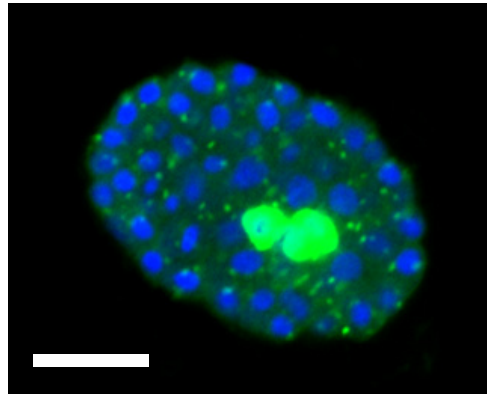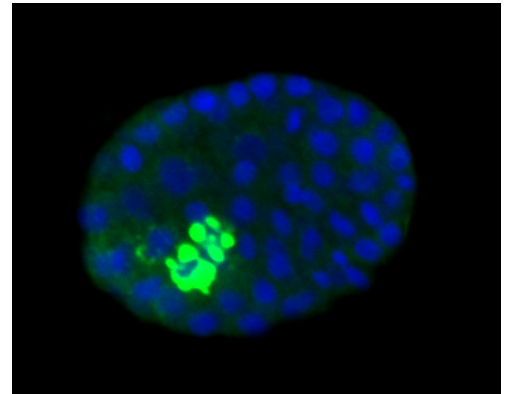

*atg-4.1*

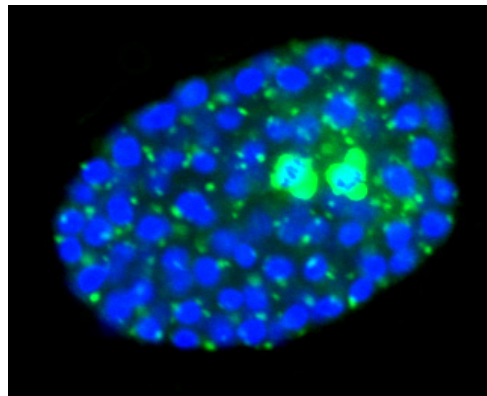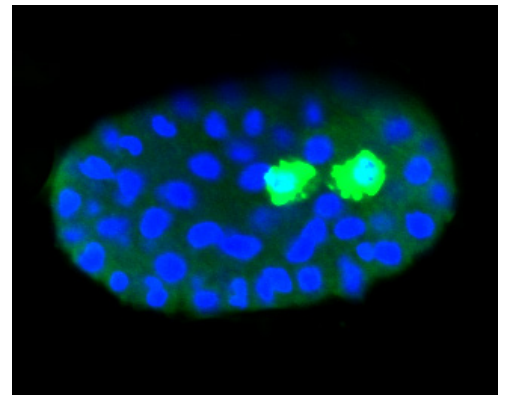

*atg-7*

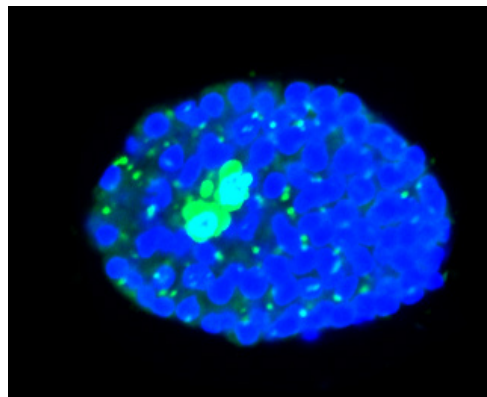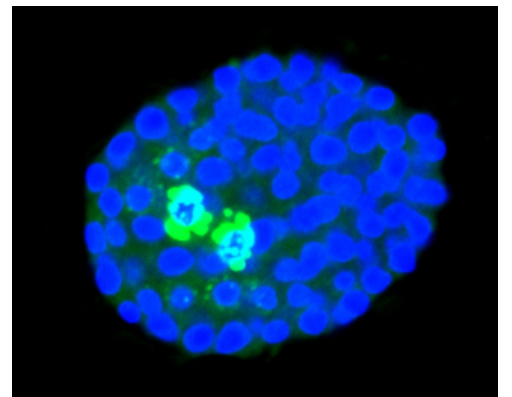

*atg-18*

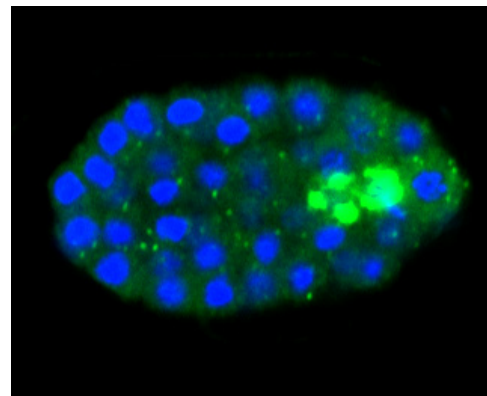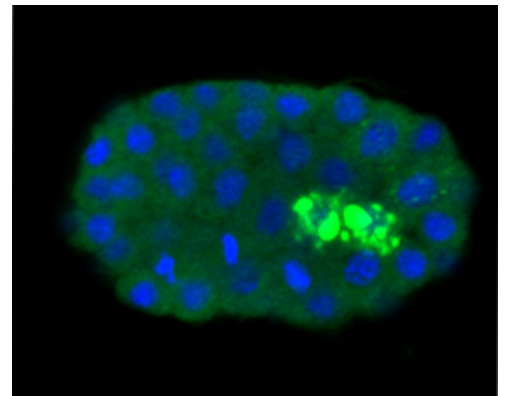

Supplement: S4 Fig — Autophagy mutants, atg-3(bp412), atg-4.1(bp501), atg-7(bp411), and atg-18(gk378), were treated or not treated with sepa-1 (M01E5.6) RNAi depletion in their P0 generation, and their F1 embryos were fixed and immunostained with anti-PGL-1 antibody (green) along with TO-PRO-3 DNA staining (blue). Note that the two blastomeres, which were immunostained strongly and consistently with anti-PGL-1 antibody with or without sepa-1 RNAi, are Z2 and Z3 embryonic germline precursor cells and not somatic blastomeres. Scale bar, 20 μm. Number of embryos examined, n ≥ 10 for respective autophagy mutants after respective RNAi treatments. (PDF) [file pgen.1008150.s008.pdf]

**A**

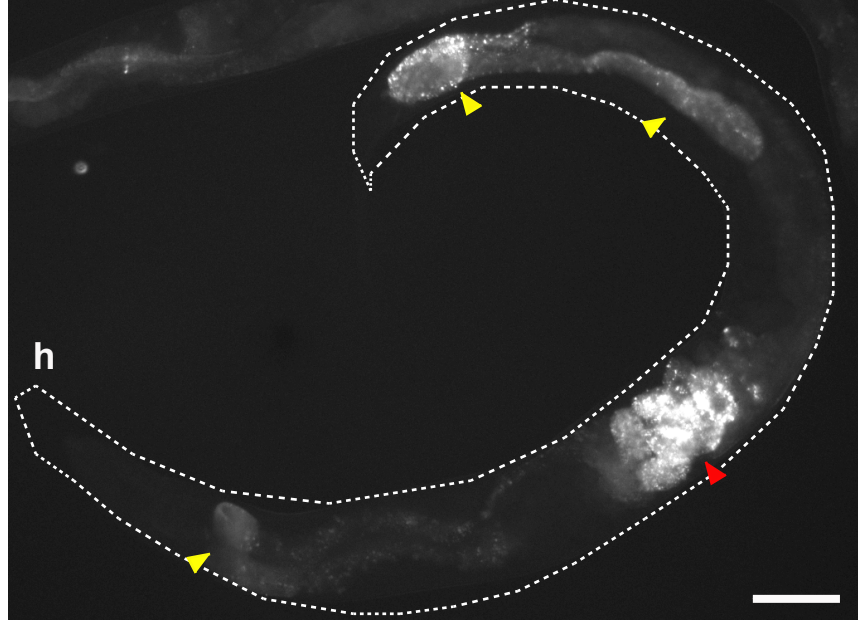

**B**

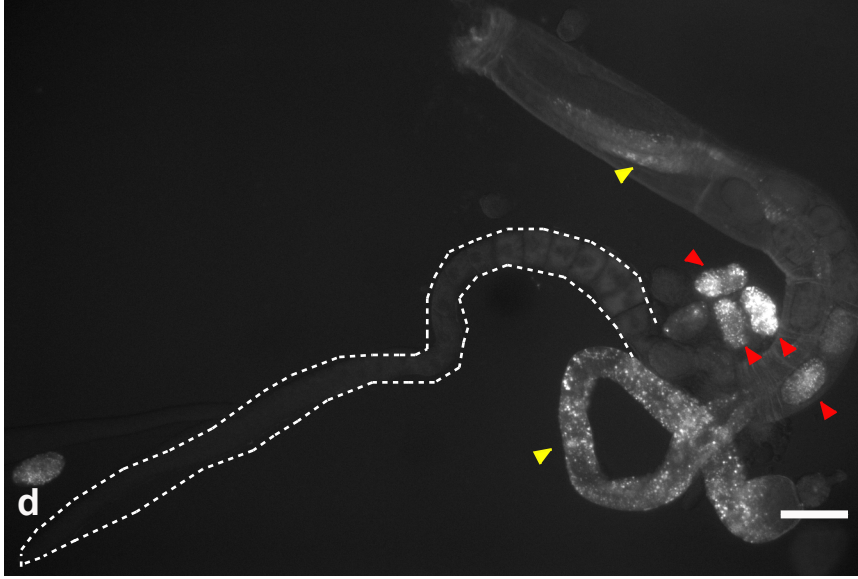

**C**

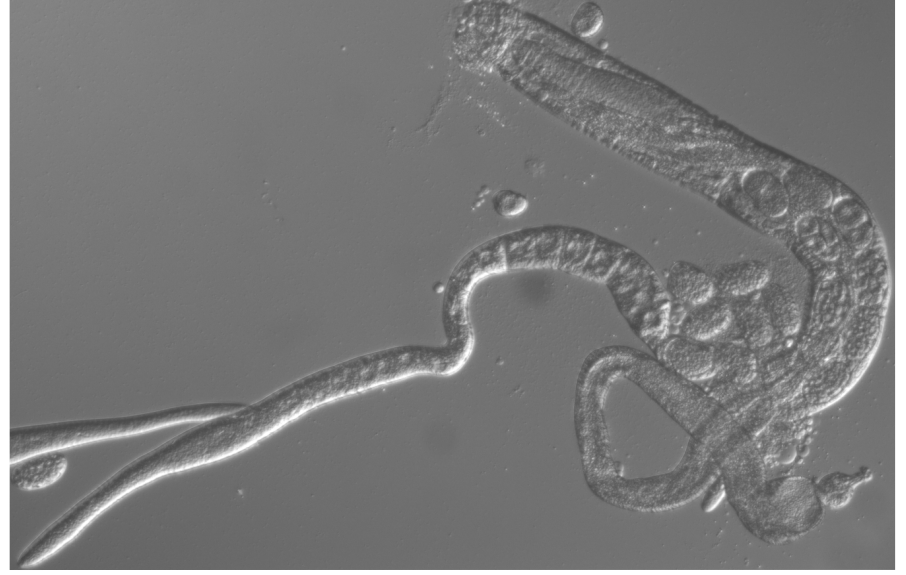

Supplement: S5 Fig — (A) A fluorescence image of an intact Psepa-1::sepa-1::GFP transgenic adult hermaphrodite. (B) A fluorescence image of a dissected Psepa-1::sepa-1::GFP transgenic adult hermaphrodite. (C) A Nomarski DIC image of (B). SEPA-1::GFP expression was observed in the anterior and posterior portions of the intestine (yellow arrowheads) and in the embryos (red arrowheads), but not in the germ cells of their gonads. h, head of the animal. d, distal end of the gonad. Scale bars, 100 μm. Number of worms examined, n = 7. (PDF) [file pgen.1008150.s009.pdf]

**A**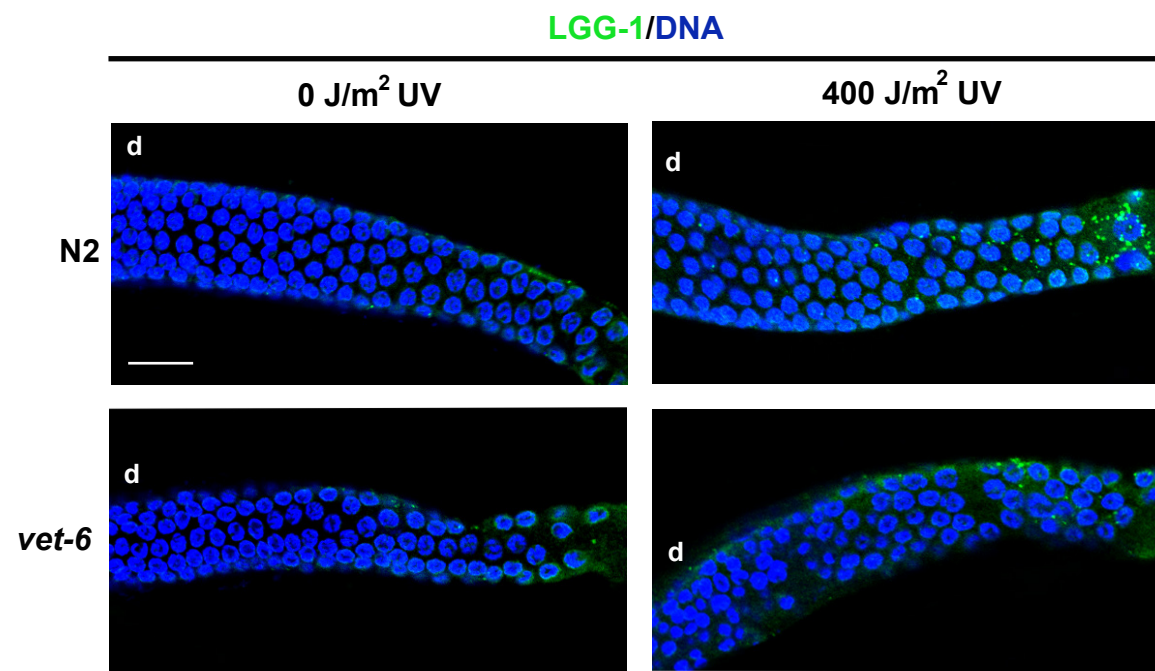**B**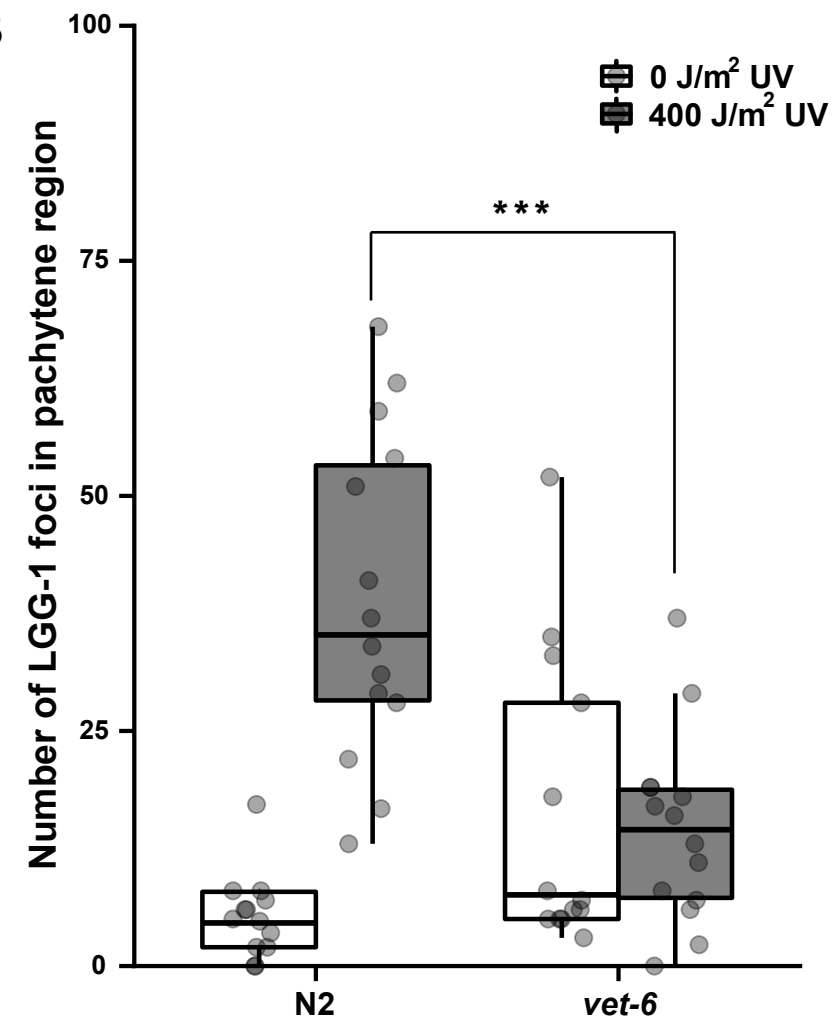

Supplement: S6 Fig — (A) N2 and vet-6(tm1226) hermaphrodites were irradiated or not irradiated with 400 J/m2 of UV at 24 h post the L4 stage, collected at 3 h after the UV irradiation, and dissected and immunostained with anti-LGG-1 antibody (green) along with DNA counterstaining (blue). Pachytene region of their gonads is shown. d, distal side of each gonad arm. Scale bar, 20 μm. (B) Box-and-whisker plots depicting the number of LGG-1 foci formed in the pachytene region of N2 and vet-6(tm1226) hermaphrodite gonads with or without 400 J/m2 of UV irradiation. The box-and-whisker plots are drawn as in S1C Fig. Number of analyzed gonads, n ≥ 10 for both of the strains in respective conditions. Statistical significance was calculated using Student’s t-test. ***, p < 0.001. (PDF) [file pgen.1008150.s010.pdf]
