# Supplementary material for: Autophagy of germ-granule components, PGL-1 and PGL-3, contributes to DNA damage-induced germ cell apoptosis in C. elegans
Source: PLoS Genet. 2019 May 24;15(5):e1008150. doi: 10.1371/journal.pgen.1008150 (PMC6534287; doi:10.1371/journal.pgen.1008150)
Supplement: S7 Fig — (A) Wild-type N2, cep-1(gk138), and egl-1(n487) hermaphrodites were dissected and immunostained with anti-LGG-1 antibody (green) along with DNA counterstaining (blue). Pachytene region of their gonads is shown. d, distal side of each gonad arm. Scale bar, 20 μm. (B) Box-and-whisker plots depicting the number of LGG-1 foci formed in the pachytene region of gonad arms from N2, cep-1(gk138), and egl-1(n487) adult hermaphrodites without UV irradiation. The box-and-whisker plots are drawn as in S1C Fig. Number of analyzed gonads, n ≥ 10 for respective strains. Statistical significance was calculated using Student’s t-test. n.s., p > 0.05 against N2 gonads. (PDF) [file pgen.1008150.s011.pdf]

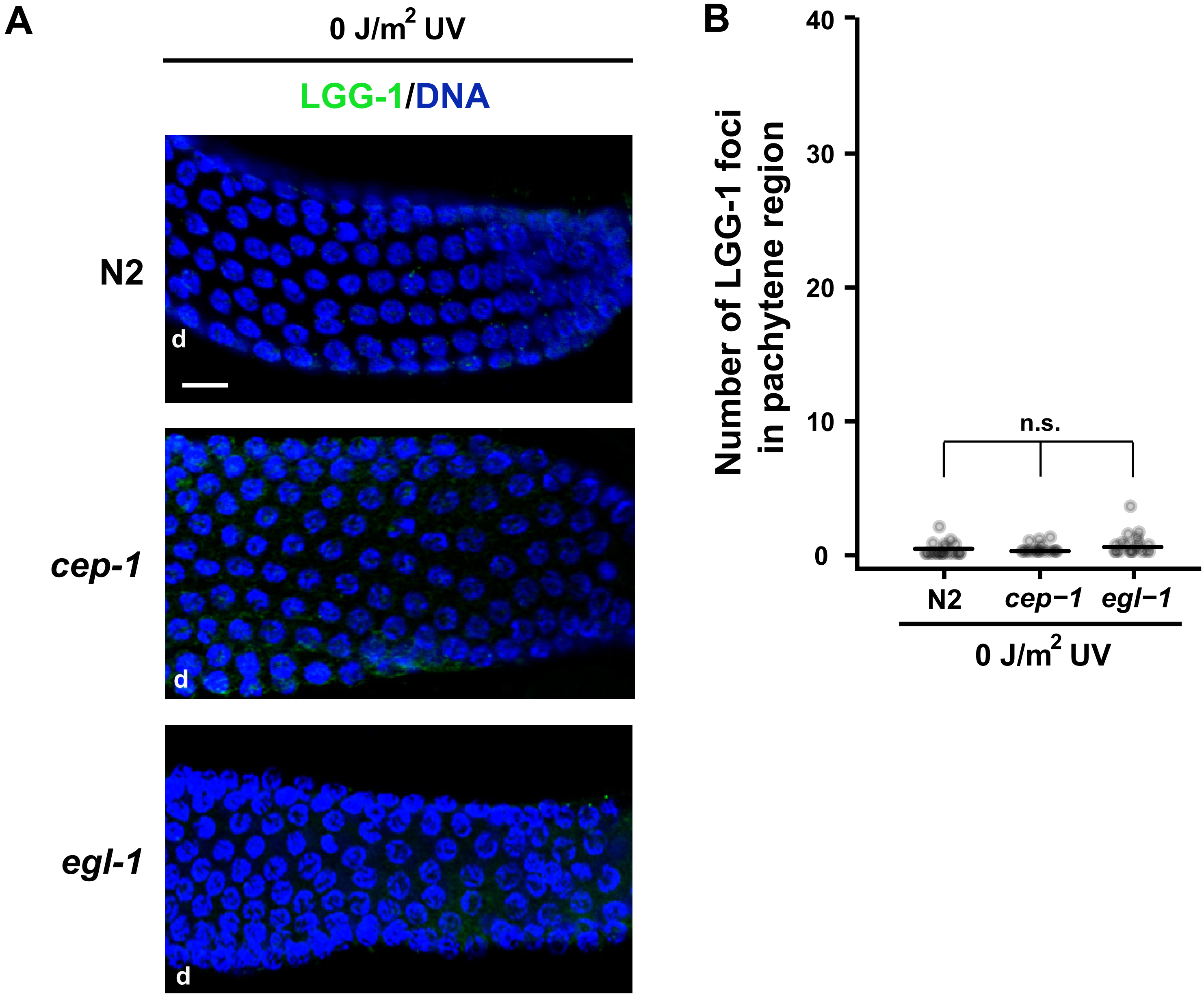

A
0 J/m2 UV
LGG-1/DNA
N2
cep-1
egl-1
d
d
d
B
Number of LGG-1 foci in pachytene region
40
30
20
10
0
n.s.
N2
cep−1
egl−1
0 J/m2 UV
